# Childhood urbanicity is associated with emotional episodic memory-related striatal function and common variation in *NTRK2*

**DOI:** 10.1186/s12916-024-03365-4

**Published:** 2024-04-02

**Authors:** Xiao Zhang, Yuyanan Zhang, Hao Yan, Hao Yu, Dai Zhang, Venkata S. Mattay, Hao Yang Tan, Weihua Yue

**Affiliations:** 1 Institute of Mental Health, National Clinical Research Center for Mental Disorders, Peking University Sixth Hospital, 51 Huayuanbei Road, Haidian District, Beijing, China; 2 NHC Key Laboratory of Mental Health (Peking University), Beijing, China; 3 Department of Psychiatry, Jining Medical University, Jining, Shandong China; 4 PKU-IDG/McGovern Institute for Brain Research of Peking University, Beijing, China; 5 Lieber Institute for Brain Development, Baltimore, MD USA; 6 Department of Neurology and Radiology, Johns Hopkins University School of Medicine, Baltimore, MD, USA; 7 Department of Psychiatry and Behavioral Sciences, Johns Hopkins University School of Medicine, Baltimore, MD USA; 8 Research Unit of Diagnosis and Treatment of Mood Cognitive Disorder (2018RU006), Chinese Academy of Medical Sciences, Beijing, China; 9 Chinese Institute for Brain Research, Beijing, China

**Keywords:** Urbanicity, Episodic memory, Striatum, Hippocampus, Genetic variant

## Abstract

**Background:**

Childhoods in urban or rural environments may differentially affect the risk of neuropsychiatric disorders, possibly through memory processing and neural response to emotional stimuli. Genetic factors may not only influence individuals’ choices of residence but also modulate how the living environment affects responses to episodic memory.

**Methods:**

We investigated the effects of childhood urbanicity on episodic memory in 410 adults (discovery sample) and 72 adults (replication sample) with comparable socioeconomic statuses in Beijing, China, distinguishing between those with rural backgrounds (resided in rural areas before age 12 and relocated to urban areas at or after age 12) and urban backgrounds (resided in cities before age 12). We examined the effect of childhood urbanicity on brain function across encoding and retrieval sessions using an fMRI episodic memory paradigm involving the processing of neutral or aversive pictures. Moreover, genetic association analyses were conducted to understand the potential genetic underpinnings that might contribute to memory processing and neural mechanisms influenced by early-life urban or rural environments.

**Results:**

Episodic memory retrieval accuracy for more difficult neutral stimuli was similar between those with urban and rural childhoods, whereas aversive stimuli elicited higher retrieval accuracy in the urban group (*P* = 0.023). For aversive stimuli, subjects with urban childhood had relatively decreased engagement of the striatum at encoding and decreased engagement of the hippocampus at retrieval. This more efficient striatal encoding of aversive stimuli in those with urban childhoods was associated with common variation in neurotrophic tyrosine kinase receptor type 2 (*NTRK2*) (right striatum: *P* = 1.58×10^−6^). These findings were confirmed in the replication sample.

**Conclusions:**

We suggest that this differential striatal processing of aversive stimuli observed in individuals with urban or rural childhoods may represent mechanisms by which childhood urbanicity may affect brain circuits, heightening behavioral responses to negative stressors associated with urban environments. *NTRK2*-associated neural processes in the striatum may play a role in these processes.

**Supplementary Information:**

The online version contains supplementary material available at 10.1186/s12916-024-03365-4.

## Background

Episodic memory is the memory of autobiographical events that occur at a particular time and place, engaging the medial temporal lobe (MTL) and prefrontal cortex (PFC) [1–3]. Episodic memory can be categorized into emotional and non-emotional stimuli [4]. Aversive content and emotional arousal have consistently been shown to increase memory of previously encoded items compared to neutral ones. The hippocampus plays an important role in processing negative or stressful information. Therefore, inhibiting negative memory engrams in the hippocampus could be a novel therapeutic approach for treating the cognitive symptoms of depression [5]. The striatum is also important for episodic memory formation, and successful memory is associated with greater activity in the striatum during encoding [6].

Deficits in both emotional and non-emotional episodic memories have been observed in various neuropsychiatric disorders [6]. In a neutral encoding task, patients with schizophrenia and their healthy siblings showed reduced parahippocampal activation and hippocampal-parietal coupling during the encoding of neutral stimuli compared with normal control participants [7]. Patients with depression demonstrated impairments in selecting relevant positive information [8]. Individuals with anxiety disorders showed enhanced memory for threatening disorder-related material [9], but impaired memory for neutral information [10]. In addition, police officers with post-traumatic stress disorder (PTSD) exhibited smaller hippocampal volumes, higher cortisol levels, and memory impairments [11]. Other studies have suggested that the amygdala has heightened responsivity in symptomatic states of PTSD during the processing of trauma-unrelated affective information, whereas the responsivity of the medial prefrontal cortex (MPFC) is inversely associated with PTSD symptom severity [12].

Urbanicity is a major socio-ecological change especially in this century. By 2050, the urban population is expected to increase to 66%, whereas the rural population is expected to decline [13]. Previous studies have suggested that the environment during childhood affects brain development [14]. Urban childhood was negatively correlated with the gray matter volume (GMV) of the MPFC in developed and developing countries [15, 16], while positively correlated with the GMV of the dorsal lateral prefrontal cortex (DLPFC) only in developing countries [16]. Meanwhile, activation of the pregenual anterior cingulate cortex (pACC) in a social stress task was affected by childhood urbanicity [17] and interacts with polygenic risk score to affect brain activation under social-stress working memory task [18]. Different urban and rural childhood environments can affect early memory development. Among children aged 10 to 13 years, those with early rural childhoods were more likely to remember information about social interactions, while those with early urban childhoods were more likely to report individual memories, and these memories appeared to contain more words [19]. However, the impact of childhood urbanicity on the neural correlates of episodic memory remains poorly understood.

Although urban environments can facilitate a higher average quality of life [20], they may also be accompanied by an increased risk of neuropsychiatric disorders, including depression, autistic spectrum disorders, and psychosis [21–24]. Additionally, a higher genetic risk for psychiatric disorders has been reported to affect individuals’ choice of residence [25]. From twin studies, living in an urban environment is itself partially heritable [26]. Although the idea that childhood environment is heritable may seem counterintuitive, work on behavioral genetics has long documented the heritability of many exposures perceived as environmental [27]. This heritability is referred to as gene–environment correlation (rGE), and potential rGE mechanisms may be posited to explain the heritability of childhood environments [28]. One such mechanism is “active” rGE, where individuals with genetic variants associated with certain behavioral phenotypes may be more prone to selecting adverse situations. For instance, genetic factors related to individuals’ nature experiences may lead children who experience more nature to benefit more from it [29]. Therefore, we hypothesized that differences in episodic memories affected by urbanicity may have genetic influences, as reflected in brain activity. The significant loci found in genome-wide association studies may provide clues about the mechanism of partial heritability on the impact of urbanicity on episodic memory.

China has undergone large-scale urbanization since the 1980s, accompanied by its economic development [30]. This gave us a unique opportunity to leverage China’s recent urbanization to examine different childhood rural-urban environmental effects on brain development, which are currently poorly understood. The aim of this study was to explore how the childhood environment could affect episodic memory brain function. To achieve this, we first compared aversive or neutral episodic memory performance across participants from rural and urban childhood environments. Second, we explored the effect of urbanicity on brain activity and investigated the correlation between brain activity and performance. Third, considering that the mechanism of urbanization effects on the brain activity associated with episodic memories remains unknown and may be influenced by genetic variation, we also conducted genome-wide genotyping of the discovery sample. Subsequently, we performed a genome-wide association study with urban-rural differences in brain activity as the dependent variable to explore the potential genetic effects. Participants in this study were balanced between their current urban environments and genetic backgrounds [18], thus maximizing the effect of childhood urban versus rural upbringing.

## Methods

### Participants

A total of 522 healthy subjects were recruited from the local community using social media and posters and 410 subjects were included in this study, all of whom had been living in Beijing for at least 1 year and had different childhood urbanicities. In this study, we divided subjects into two main groups: the urban group (*N*=220) comprised adult subjects who lived in cities before the age of 12 years, while the rural group (*N*=190) comprised those who only moved to cities after the age of 12 years. We also conducted analysis with an increased grouping resolution of four groups (born in and continue to live in cities, born in rural areas, and lived in cities since before 12 years, born in rural areas and lived in cities since 12–18, born and lived in rural areas for >18 years since birth) and utilizing urbanicity scores [17]. The detailed recruitment methods are described in the Additional file 1: Supplementary Methods [31–33].

### Episodic memory paradigm

**Fig. 1:**
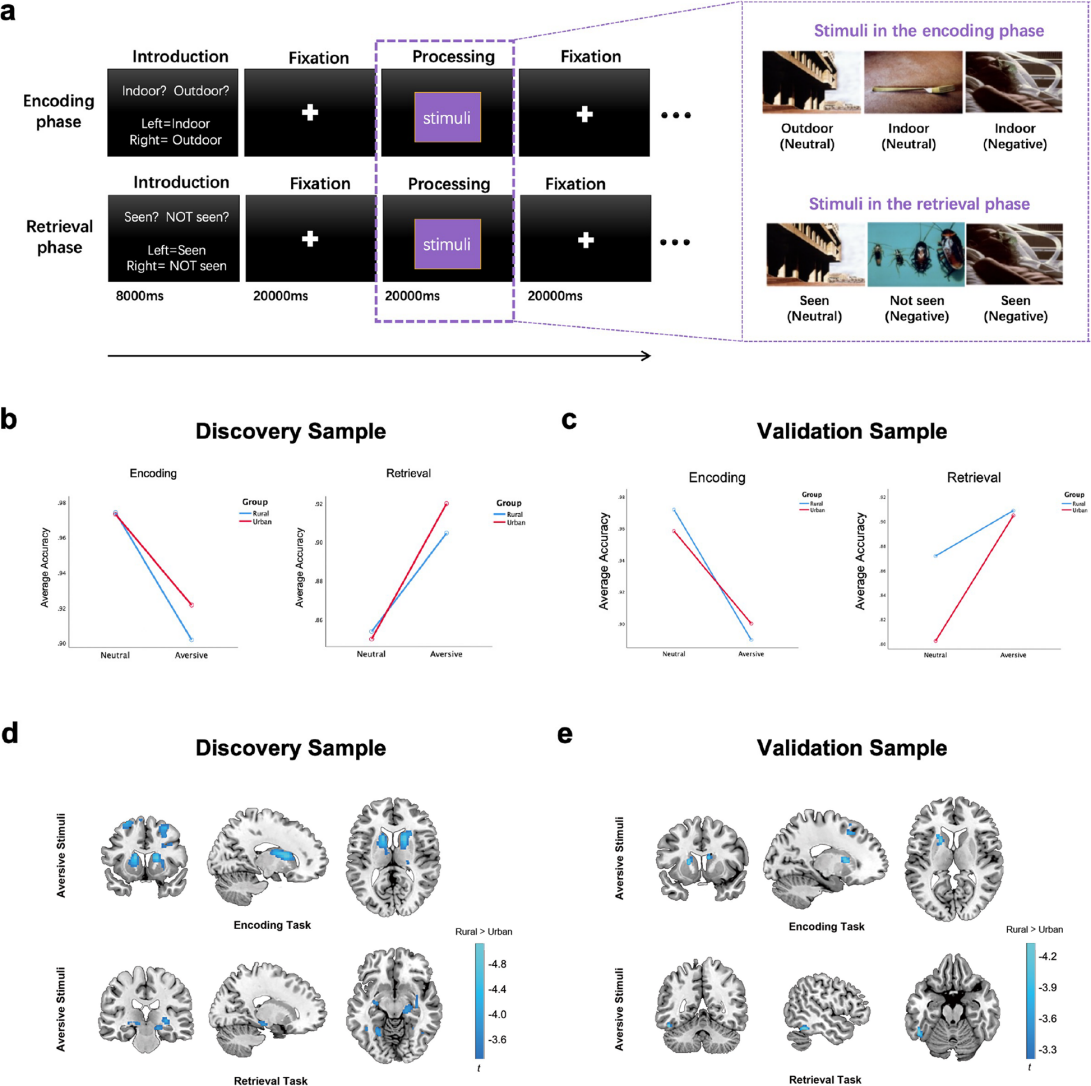
Episodic memory paradigm, behavioral performance, and brain activation across rural and urban groups in the discovery and replication samples. **a** Episodic memory paradigm. **b** The interaction effect of behavioral performance during encoding and retrieval sessions in the discovery sample. **c** The interaction effect of behavior performance during encoding and retrieval sessions in the replication sample. **d** Discovery sample: During the encoding session, the rural group engaged more activation at bilateral caudate and putamen, bilateral middle frontal gyrus under aversive stimuli. During the retrieval session, the rural group engaged more activation at bilateral hippocampus, right amygdala, left thalamus, and fusiform under aversive stimuli (whole brain cluster-wise FWE-corrected *P* < 0.05). **e** Replication Sample: During the encoding session, the rural group engaged activation at bilateral caudate, left middle and superior frontal gyrus, and anterior cingulate cortex under aversive stimuli. During the retrieval session, the rural group also engaged activation in left inferior temporal gyrus, thalamus, and bilateral middle frontal gyrus than the urban group (*P* < 0.001 uncorrected, *k* > 30)

We performed an episodic memory task with encoding and retrieval sessions of aversive and neutral scenes selected from the International Affective Picture System (Fig. 1a) [34]. This task has been shown to reliably engage the hippocampus and the temporal, parietal, and frontal cortices in healthy volunteers [7, 35–38]. The detailed task design is provided in the Additional file 1: Supplementary Methods. The scenes were shown in a block design paradigm with two blocks of aversive/neutral compared with the resting state. The encoding blocks showed 6 scenes of similar valence (neutral or aversive) pictures serially to the participants for 3 s each, and the participants answered whether the pictures were “indoor” or “outdoor.” Subsequently, the retrieval blocks showed six scenes of similar valence (neutral or aversive) pictures serially to the participants for 3 s each, half of which were pictures that they had seen during the encoding session. The participants answered whether the scenes are “old” or “new.” During the resting blocks, participants were asked to observe a fixation cross presented at the center of the screen for 18 s, which was treated as the baseline in the functional magnetic resonance imaging (fMRI) analyses.

### Behavior analysis

Accuracy during encoding task was calculated as the percentage of correct responses in the total choice including the indoor and outdoor conditions. Accuracy during retrieval was calculated as the percentage of correct responses and d-primes [39], in which d-prime represents the sensitivity of participants’ responses in the signal detection task. As encoding and retrieval represent different episodic memory processes, we performed an ANOVA to explore the main and interaction effects of urbanicity (rural or urban) and valence (aversive or neutral) and further conducted a simple effect analysis separately. We compared behavioral data between the rural and urban groups using an independent sample *t*-test.

### fMRI acquisition and statistical analysis

Bold fMRI was performed on a 3.0-T General Electric Discovery MR750 scanner, and standard preprocessing analysis was performed using MATLAB and SPM12 (www.fil.ion.ucl.ac.uk/spm); the detailed scanning parameters and analysis protocols are described in the Additional file 1: Supplementary Methods. For both the encoding and retrieval sessions, we performed a first-level analysis using the general linear model (GLM) with six head motions as covariates of no interest. Individual *t*-contrast maps were generated for both sessions with the following contrasts of interest: aversive > baseline and neutral > baseline. A one-sample *t*-test was performed to examine the whole sample activation information in both the encoding and retrieval tasks under different valences, with age as the nuisance covariate. We tested the urbanicity effect bidirectionally using age and sex as nuisance covariates.

To assess the brain regions associated with recognition accuracy, regression analyses were performed across individual activation maps and recognition accuracy in both encoding and retrieval, with age and sex as nuisance covariates. Estimates of the weighted beta parameters were extracted from significant voxels within regions of interest (ROIs) that showed significant urban-rural differences using the MARSBAR toolbox (http://marsbar.sourceforge.net) and exported to R (https://www.r-project.org/) to calculate the correlation analysis. Statistical thresholds for the imaging analyses of the discovery sample were set at *P* < 0.05, family-wise error rate (FWE) cluster-wise, with *P* < 0.001 voxel-wise corrected for the whole brain.

### Replication in an independent sample of healthy adults

To test whether the differences between urban and rural groups under aversive stimuli in the encoding and retrieval task that survived in the whole-brain cluster-wise FWE-corrected were robust, we recruited another independent sample of healthy subjects from urban and rural childhood environments from the local community using social media and posters (*N*=72, 35 subjects who lived in cities before the age of 12 years and 37 subjects from rural areas who only moved to cities after the age of 12 years). They completed Blood Oxygenation Level Dependent (BOLD) fMRI with a 3.0-T General Electric Discovery MR750 scanner (the same model scanner as that in the discovery sample) at the Neuroimaging Center, Peking University Sixth Hospital, using consistent parameters, and data were analyzed in the same way as the previous dataset. The statistical threshold was set at *P* < 0.001, uncorrected, with a cluster extent threshold of *k* > 30 because of the limited number of subjects.

### Genetic association analysis

DNA collection and genome-wide genotyping of the discovery sample are described in Additional file 1: Supplementary Methods. Principal component analysis (PCA) was performed to verify the genetic backgrounds of the rural and urban groups (Additional file 1: Figure S1). Finally, nine participants were excluded after quality control. A total of 4,388,740 variants across 401 individuals were included in the genetic association analysis. Logistic regression under an additive genetic model was used to evaluate the associations between the allele dosages and the urban-rural differences in activity that survived at *P* < 0.05, FWE cluster-wise with *P* < 0.001 voxel-wise corrected for the whole brain in the encoding and retrieval task in PLINK v1.90. Age, sex, educational level, and 10 principal components were entered as covariates. The threshold was set at *P* < 5×10^−8^ to reveal significant loci, and exploratory *P* < 5×10^−6^ to reveal suggestive results.

## Results

### Demographics

In the discovery sample, participants with childhood urbanicity were slightly younger than that with rural childhood (Table 1). Both groups were currently living in Beijing and were not different in sex distribution, educational, and occupational levels. They were genetically homogeneous, with no significant differences between the first 10 principal components from the genome-wide genotyping (Additional file 1: Figure S1). In the replication sample, the participants with childhood urbanicity were younger. Similar to the discovery sample, no significant differences in gender distribution, educational level, or population stratification were observed between the urban and rural groups (Table 2).

**Table 1:** Demographical and performance data of the discovery sample

Characteristic	Rural group (Mean, SD)	Urban group (Mean, SD)	***F/χ***^**2**^	***P***
Age, years	25.8 (3.55)	24.5 (4.03)	3.244	0.001**
Age range	18–40	18–43	-	-
Sex, M/F, No.	100:90	103:117	1.378 ^a^	0.240
Sex, M%	52.6%	46.8%	-	-
Education, years	17.03 (2.56)	16.67 (2.33)	1.504	0.838
**Encoding neutral performance**				
Accuracy, % correct	97.41 (3.41)	97.31 (4.78)	0.063	0.802
RT, ms	855.73 (136.14)	813.74 (146.27)	7.277	0.007**
**Encoding aversive performance**				
Accuracy, % correct	90.17 (7.21)	92.16 (7.72)	7.552	0.006**
RT, ms	1027.07 (180.52)	1001.70 (183.71)	1.172	0.280
**Retrieval neutral performance**				
Accuracy, % correct	85.37 (8.16)	84.98 (8.12)	0.814	0.367
RT, ms	1070.72 (149.68)	1065.71 (159.85)	0.001	0.979
d prime	2.44 (0.73)	2.40 (0.71)	0.922	0.337
**Retrieval aversive performance**				
Accuracy, % correct	90.46 (6.74)	92.00 (6.84)	4.909	0.027*
RT, ms	1110.21 (160.40)	1089.07 (167.14)	0.796	0.373
d prime	2.93 (0.72)	3.10 (0.71)	5.303	0.020*

^a^ These variables were compared by using *χ*^2^ tests

*M*, male; *F*, female; *RT*, reaction time; **P <0.01, *P <0.05

Age, sex, and educational level were used as covariates when comparing behavior results

**Table 2:** Demographical and performance data of the replication sample

Characteristic	Rural group (Mean, SD)	Urban group (Mean, SD)	***F/χ***^**2**^	***P***
Age, years	25.9 (3.87)	23.1 (2.0)	3.826	<0.001***
Age range	19–33	18–26	-	-
Sex, M/F, No.	18:19	16:19	0.062 a	0.803
Sex, M%	48.6%	45.7%	-	-
Education, years	17.22 (2.19)	16.40 (1.72)	1.754	0.084
**Encoding neutral performance**				
Accuracy, % correct	97.18 (3.68)	95.83 (9.95)	1.262	0.265
RT, ms	795.99 (123.05)	809.31 (114.40)	0.657	0.420
**Encoding aversive performance**				
Accuracy, % correct	88.96 (6.60)	90.00 (8.94)	0.913	0.343
RT, ms	959.20 (158.47)	996.19 (168.64)	0.647	0.424
**Retrieval neutral performance**				
Accuracy, % correct	87.16 (8.12)	80.24 (14.27)	9.937	0.002**
RT, ms	1015.03 (152.02)	999.92 (139.42)	0.981	0.325
d prime	2.86 (0.42)	2.58 (0.80)	3.085	0.084
**Retrieval aversive performance**				
Accuracy, % correct	90.88 (7.40)	90.48 (5.85)	0.061	0.806
RT, ms	1080.53 (167.57)	1011.60 (140.98)	5.481	0.022*
d prime	2.93 (0.59)	2.96 (0.50)	0.079	0.780

^a^ These variables were compared by using *χ*^2^ tests

*M*, male; *F*, female; *RT*, reaction time; **P <0.01, *P <0.05

Age, sex, and educational level were used as covariates when comparing behavior results

### Behavioral results

#### Discovery sample

In the encoding task, two-way repeated-measures ANOVA with age, gender, and educational level as covariates showed a significant interaction effect between urbanicity and stimulus valence (aversive or neutral) [partial eta squared (*η*^2^_*p*_) = 0.021, *P* = 0.003, Figure 1b]. The accuracy of the aversive task was generally lower than that of the neutral task in both groups (*P* < 0.001); however, this effect was more pronounced in the rural group (rural group: *η*^2^_*p*_ = 0.316; urban group: *η*^2^_*p*_ = 0.210; considering effect size *η*^2^_*p*_ >0.14 as large; around 0.06 are medium; and <0.01 small [40]). Simple effect analysis showed that the urban group exhibited significantly higher accuracy than the rural group for aversive stimuli (*η*^2^_*p*_ = 0.018, *P* = 0.006), but not for neutral stimuli (*η*^2^_*p*_ = 0.0002, *P* = 0.802, Table 1).

In the retrieval task, there was a significant interaction effect between urbanicity and valence (*η*^2^_*p*_ = 0.012, *P* = 0.015, Figure 1b). The accuracy of the aversive task was higher than that of the neutral task in both groups (*P* < 0.001); however, this effect was more pronounced in the urban group (rural group: *η*^2^_*p*_ = 0.127; urban group: *η*^2^_*p*_ = 0.261). The urban group exhibited significantly higher accuracy than the rural group for aversive stimuli (*η*^2^_*p*_ = 0.012, *P* = 0.027), but not for neutral stimuli (*η*^2^_*p*_ = 0.002, *P* = 0.367, Table 1).

#### Replication sample

In the encoding task, two-way repeated-measures ANOVA with age, gender, and educational level as covariates showed there were no significant differences between the rural and urban groups in terms of accuracy (Table 2), and there was no significant interaction effect between urbanicity and valence (*η*^2^_*p*_ = 0.036, *P* = 0.116, Figure 1c). The accuracy of the aversive task was generally lower than that of the neutral task in both groups; however, this effect was more pronounced in the rural group (rural group: *η*^2^_*p*_ = 0.292, *P* < 0.001; urban group: *η*^2^_*p*_ = 0.103, *P* = 0.007).

In the retrieval task, there was a significant interaction effect between urbanicity and valence (*η*^2^_*p*_ = 0.111, *P* = 0.005, Figure 1c). The accuracy of the aversive task was higher than that of the neutral task in urban groups (*η*^2^_*p*_ = 0.313, *P* < 0.001), but not in the rural group (*η*^2^_*p*_ = 0.026, *P* = 0.184). The urban group exhibited significantly lower accuracy than the rural group for neutral stimuli (*η*^2^_*p*_ = 0.129, *P* = 0.002), but not for aversive stimuli (*η*^2^_*p*_ = 0.001, *P* = 0.806, Table 2).

### fMRI task activation

During the encoding and retrieval sessions under both neutral and aversive stimuli, regions in the DLPFC, occipital visual cortex, parts of the temporal and parietal lobes, hippocampus, striatum, and amygdala exhibited robust engagement. Conversely, decreased engagement was observed in parts of the MPFC, posterior cingulate cortex (PCC), insula, and precuneus (Additional file 1: Figure S2, Table S1 and Table S2, whole-brain FWE-corrected *P* <0.05, *k* > 100).

### Effects of valence

To investigate the effects of stimulus valence on the neural circuitry of declarative memory, the aversive condition was compared with the neutral condition across encoding and retrieval sessions. During the encoding session, significantly greater activation in response to aversive scenes was observed in many brain regions, including the bilateral MPFC, DLPFC, precuneus, hippocampus, amygdala, striatum, fusiform, thalamus, and parts of the temporal lobe (including the temporal pole). Conversely, significantly decreased activity was observed in the bilateral insula, inferior parietal lobule, and angular gyrus (Additional file 1: Figure S2 and Table S1, whole-brain FWE-corrected *P* <0.05, *k* > 100). During the retrieval session, significantly greater activity was observed for aversive scenes in similar brain regions, with only the right superior temporal gyrus showing decreased activation compared to that of neutral stimuli (Additional file 1: Figure S2 and Table S2, whole-brain FWE-corrected *P* <0.05, *k* > 100).

### Effects of urbanicity

We explored the rural-urban difference in two contrast images (i.e., neutral-baseline and aversive-baseline) during both the encoding and retrieval sessions. During the encoding session, the rural group showed increased activation relative to the urban group in the bilateral caudate, putamen, and bilateral middle frontal gyrus under aversive stimuli (Figure 1d, Table 3, whole-brain cluster-wise FWE-corrected *P* < 0.05). Under neutral stimuli, there were no regions with differential activity that survived with FWE-corrected *P* < 0.05. Only the right caudate and putamen showed greater activation in the rural group than the urban group, while the urban group exhibited greater angular activation than the rural group (Table 3, *P* < 0.001, uncorrected, *k* >100).

**Table 3:** The effects of urbanicity in the encoding and retrieval tasks of the discovery and replication sample

Regions	Cluster size	***P***_***cluster-FWE***_	***x***	***y***	***z***	***T***	***P***_***peak-FWE***_
**Discovery sample: encoding task**							
**Aversive: rural > urban**							
R middle frontal gyrus	3021	< 0.001	24	32	40	5.08	0.005
R caudate and putamen			16	12	12	4.93	0.010
L caudate and putamen	1070	< 0.001	−14	12	12	4.91	0.011
			−14	2	16	4.67	0.028
L middle frontal gyrus	376	0.025	−28	10	64	4.45	0.066
			−24	8	54	4.16	0.180
**Neutral: rural > urban**							
R caudate and putamen	116	0.359	20	16	12	3.99	0.287
**Neutral: rural < urban**							
R angular	104	0.406	58	−60	30	3.57	0.715
**Discovery sample: retrieval task**							
**Aversive: rural > urban**							
R amygdala and hippocampus	442	0.015	34	−8	−14	4.20	0.152
			18	−24	−10	3.88	0.390
L thalamus and hippocampus	412	0.020	−24	−30	2	4.37	0.087
L middle temporal gyrus	389	0.024	−38	−58	6	4.07	0.231
L fusiform			−40	−54	−6	3.70	0.579
**Neutral: rural > urban**							
L thalamus	154	0.246	−24	−28	−2	4.21	0.142
L fusiform	119	0.351	−42	−50	−10	4.07	0.221
L fusiform	204	0.149	−26	−54	−12	4.04	0.243
**Replication sample: encoding task**							
**Aversive: rural > urban**							
L caudate and putamen	111	0.349	−18	10	10	4.29	0.325
L superior frontal gyrus	78	0.528	−22	20	46	3.96	0.623
R caudate	33	0.847	10	10	16	3.88	0.701
**Neutral: rural < urban**							
Parietal lobe	432	0.011	−38	−28	22	5.05	0.034
Precentral gyrus	340	0.027	−28	−42	30	4.99	0.042
**Replication sample: retrieval task**							
**Aversive: rural > urban**							
L inferior temporal gyrus	59	0.653	−50	−50	−20	4.27	0.346
R middle frontal gyrus	33	0.848	30	10	50	3.89	0.711
L middle frontal gyrus	79	0.513	−26	6	46	3.88	0.721
**Neutral: rural > urban**							
L inferior temporal gyrus	41	0.789	−48	−52	−18	3.71	0.847
L fusiform	31	0.859	−34	−10	−24	3.67	0.871

*P*_*cluste-FWE*_, *P* < 0.05, whole-brain cluster-wise FWE correction

*P*_*peak-FWE*_, *P* < 0.05, whole-brain FWE correction

During the retrieval session, the rural group showed greater activation than the urban group in the bilateral hippocampus, right amygdala, left thalamus, and fusiform under aversive stimuli (Figure 1d, Table 3, whole-brain cluster-wise FWE-corrected *P* < 0.05). Under neutral stimuli, there were no regions surviving FWE-corrected *P* < 0.05. The rural group only showed higher activation of the left thalamus and left fusiform than that of the urban group (Table 3, *P* <0.001, uncorrected, *k* > 100).

Notably, similar results were observed using ANOVA with individual *t*-contrast maps when the grouping resolution was increased to four groups: those who were born in and continued to live in cities, those who have lived in cities since before age 12, those who lived in rural areas between birth and age 18, and those who lived in rural areas for ≥18 years since birth (Additional file 1: Figure S3 and S4). Additionally, similar results were obtained using urbanicity scores [17, 41] (Additional file 1: Figure S5 and S6). Notably, participants exhibited progressive weaker brain activations with gradual exposure to levels of urbanicity (Additional file 1: Figure S4 and S6).

### Brain-behavior correlations

**Fig. 2:**
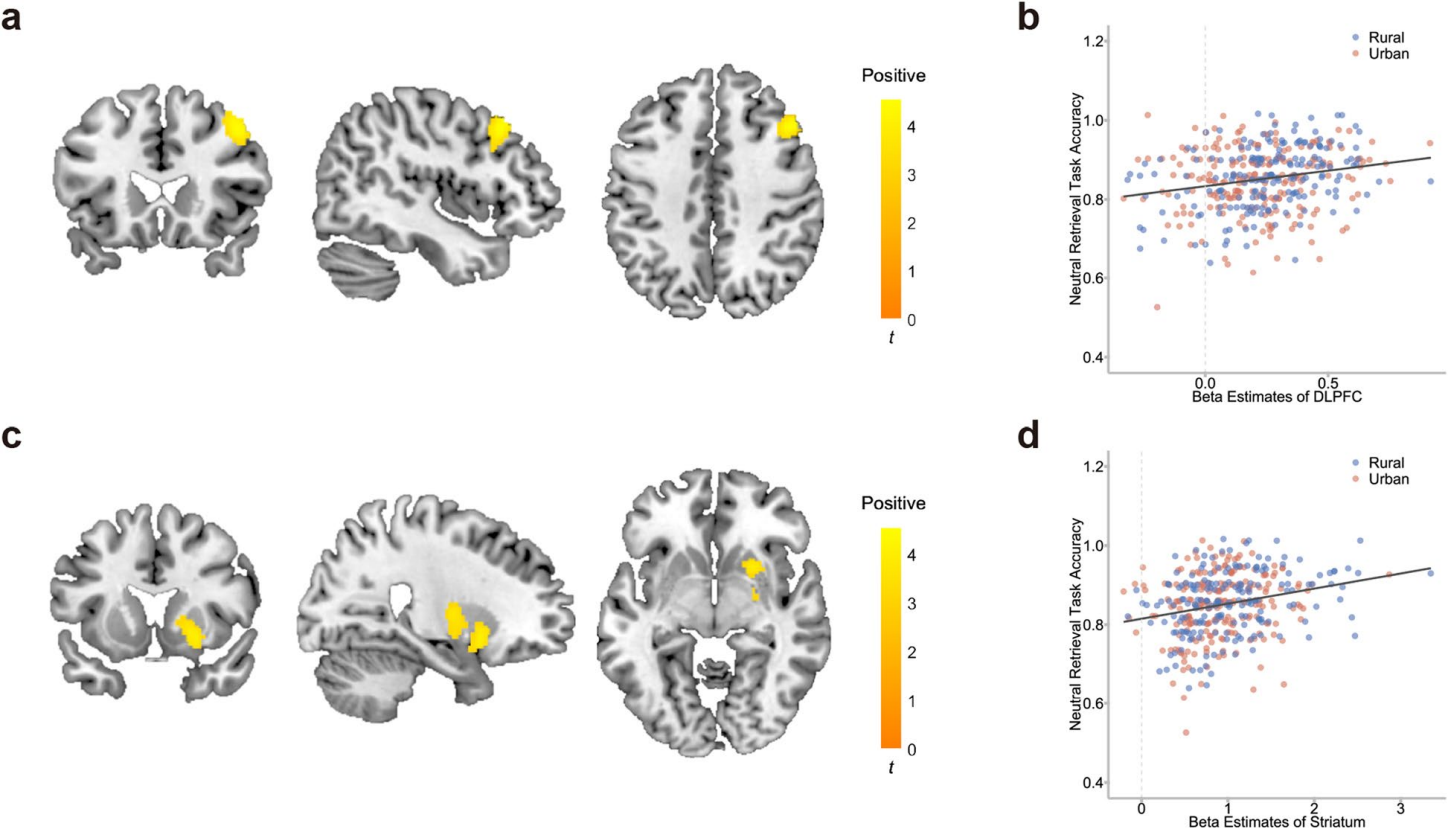
Brain-behavior relationship between encoding activation and retrieval accuracy for neutral stimuli. Encoding brain activation was associated with retrieval accuracy under neutral stimuli (*N* = 410, *P* < 0.05, whole-brain cluster-wise FWE corrected, controlled for age and gender). **a** The DLPFC activation during encoding was associated with retrieval accuracy (Peat at [44 22 42], *t* = 4.55, cluster size = 372). **b** Scatter plot showing a positive correlation between encoding DLPFC activation and retrieval accuracy (*P* < 0.001, *r* = 0.245). **c** The striatum activation during encoding was associated with retrieval accuracy (Peat at [24 12 -6], *t* = 4.11, cluster size = 579). **b** Scatter plot showing a positive correlation between encoding DLPFC activation and retrieval accuracy (*P* < 0.001, *r* = 0.213)

To assess which brain regions were associated with recognition accuracy during encoding, simple regressions were performed between encoding activation maps and recognition accuracy, with age and sex as covariates. We found that the activation of the right DLPFC (peak at [44 22 42], *t* = 4.55, cluster size = 372) and the right striatum (peat at [24 12 -6], *t* = 4.11, cluster size = 579) was associated with neutral retrieval accuracy (whole-brain cluster-wise FWE-corrected *P* < 0.05, Figure 2). Specifically, under neutral stimuli, the retrieval accuracy was positively correlated with encoding brain activations of the DLPFC (*P* < 0.001; *r* = 0.245) and the striatum (*P* < 0.001, *r* = 0.213). When we used d-prime as the dependent variable (Additional file 1: Table S5), or included age, sex, and socioeconomic status (SES) as covariates, similar results were obtained (Additional file 1: Table S6 and S7). We did not find any significant correlations under aversive stimuli.

### Genetic association

In the genome-wide association study, we extracted the average BOLD response of the most differentially activated urban-rural regions during the encoding and retrieval tasks as dependent variables. By selecting an ROI in each representative brain region, a total of 8 ROIs were drawn centered on the peak activation difference, with a 6-mm radius. These ROIs were as follows: for the encoding task, the middle frontal gyrus (peak at [24 32 40]), the right striatum (peak at [16 12 12]), the left striatum (peak at [-14 12 12]); the middle frontal gyrus (peak at [-28 10 64]); for the retrieval task, the amygdala (peak at [34 -8 -14]), the hippocampus (peak at [18 -24 -10]), the thalamus (peak at [-24 -30 2]), and the middle temporal gyrus (peak at [-38 -58 6]) (Table 3). The time series of each voxel within the ROIs were extracted and the average BOLD response obtained. We identified an exploratory single-nucleotide polymorphism (SNP) located within genes with minor allele frequency > 0.10, and |Beta| > 0.10 in the discovery sample.

The results revealed that rs7042458, an intron of the neurotrophic tyrosine kinase receptor type 2 (*NTRK2*) gene, was correlated with the BOLD response in the bilateral striatum under aversive stimuli during the encoding task (right striatum: *P* = 1.58×10^−6^, Beta = −0.1679; left striatum: *P* = 1.33×10^−6^, Beta = −0.1639, Figure 3). AA homozygotes (*n*=306) showed significantly higher activity in the bilateral striatum than AT/TT heterozygotes (*n*=95). Furthermore, the chi-square test showed that there were more individuals with the T-carrier genotype in the urban group (*χ*^2^ = 4.039, *P* = 0.044). Public expression data from the Brainace Database indicated that the common *NTRK2* rs7042458 variant affected gene expression in the putamen and frontal cortex, with the AT/TT group exhibiting higher expression (Additional file 1: Figure S7). The explained variance (adjusted R^2^) of the average BOLD response of the right striatum was increased from 5.23% (including genetic variant) to 9.59% (including both genetic variant and urbanicity). The 4.36% variance was still explained by urbanicity if genetic variants were used as predictors. The explained variance (adjusted R^2^) of the average BOLD response of the left striatum was increased from 4.23 to 8.70%. The 4.47% variance was still explained by urbanicity if genetic variants were used as predictors. Furthermore, rs9320231 (*P* = 3.46×10^−6^, Beta = −0.1846,) located within the Scm Polycomb Group Protein Like 4 (*SCML4*) gene, and rs1562086 (*P* = 3.79×10^−6^, Beta = −0.2686), located within the Alpha-1,6-Mannosylglycoprotein 6-Beta-N-Acetylglucosaminyltransferase B (*MGAT5B*) gene, were correlated with the BOLD response of the right middle frontal gyrus under aversive stimuli during the encoding task.

**Fig. 3:**
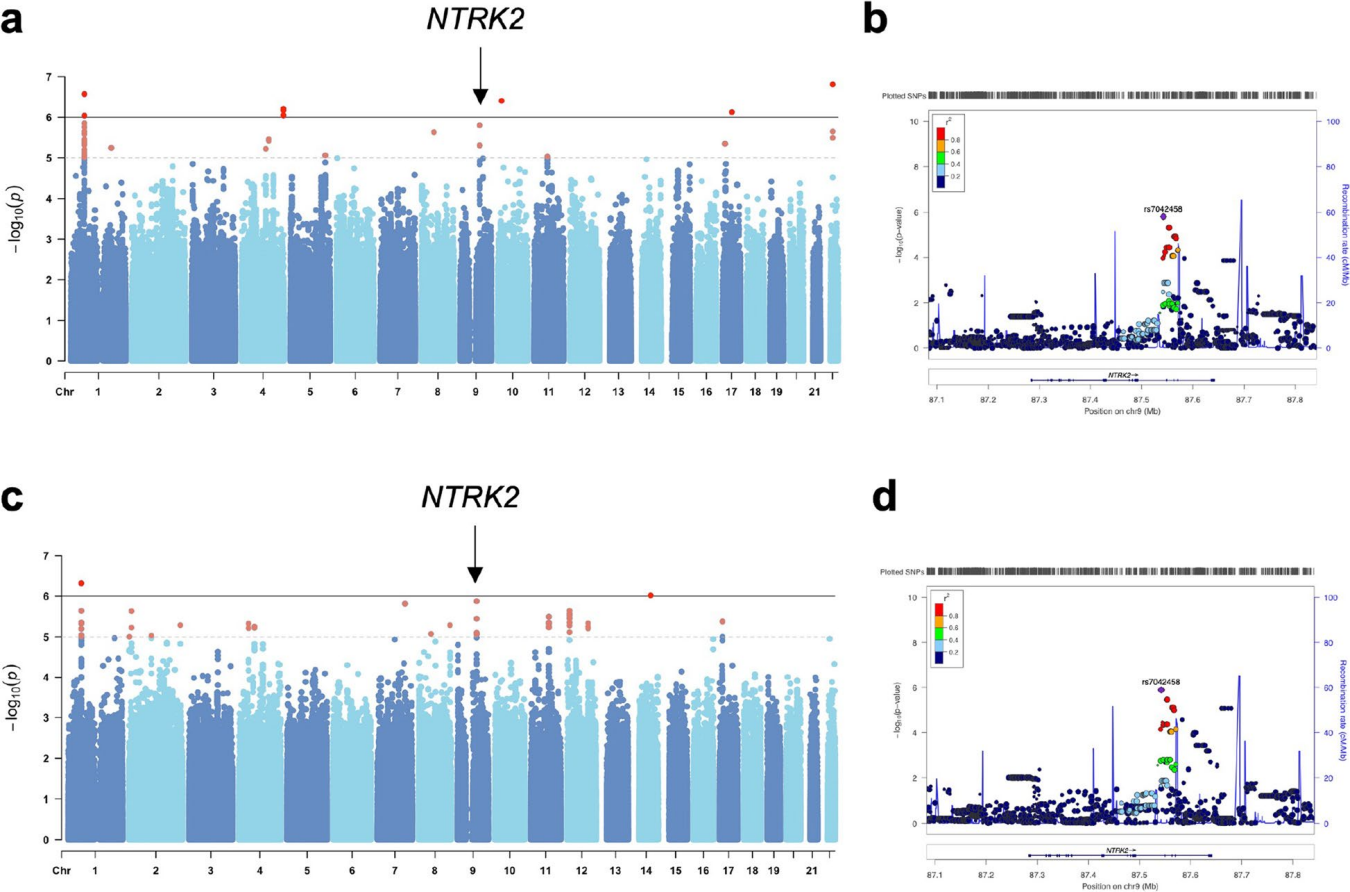
Striatal emotional encoding function was associated with the common variant in *NTRK2*. **a, b** Rs7042458, an intron of neurotrophic tyrosine kinase receptor type 2 (*NTRK2*) gene, was correlated with BOLD response of urban-rural different right striatum under aversive stimuli during encoding task (*P* = 1.58×10^−6^, Beta = −0.1679). **c, d** Rs7042458 was also associated with BOLD response of urban-rural different left striatum under aversive stimuli during encoding task (*P* = 1.33×10^−6^, Beta = −0.1639)

Under aversive stimuli in the retrieval task, rs6442936, an intron of the ADP ribosylation factor like GTPase 8 B (*ARL8B*) gene involved in lysosomal function, was significantly correlated with the BOLD response in the right amygdala (*P* = 3.52×10^−9^, Beta = 0.3796) in a small number of participants with a CG genotype (17/397). Rs423158 (*P* = 3.84×10^−6^, Beta = 0.1026), located within the Solute Carrier Family 25 member 18 (*SCL25A18*) gene, was also correlated with the BOLD response of the right amygdala. Rs71471176 (*P* = 3.44×10^-6^, Beta = 0.2044), located within the Glutamate Ionotropic Receptor Delta Type Subunit 1 (*GRID1*) gene, was correlated with the BOLD response in the right hippocampus. Rs152591 (*P* = 1.64×10^−6^, Beta = −0.1263), located within the Ephrin A5 (*EFN5A*) gene, was correlated with the BOLD response of the left middle temporal gyrus.

### Independent replication

We focused on replicating the contrast of interest (aversive vs. baseline) that showed significant brain activation associated with urbanicity. During the encoding session, the rural group showed higher activation compared to the urban group in the right caudate, left inferior frontal gyrus, and middle temporal gyrus in response to aversive stimuli. During the retrieval session, the rural group showed higher activation in the right caudate, medial frontal gyrus, and anterior cingulate cortex under aversive stimuli than that in the urban group (*P* < 0.001, uncorrected, *k* > 30, Figure 1e, Table 3). Although the regions did not survive with FWE-corrected *P* < 0.05 mainly due to the limited sample size, we observed a consistent role of urban-rural striatal activity difference in response to aversive stimuli during the encoding session. After extracting the replicated urban-rural striatal activity differences in the encoding task under aversive stimuli, we found that rs7042458 AA homozygotes (*n*=45) showed significantly higher activity in the right striatum than the AT/TT group (*n*=14, *P* = 0.042, with age and sex as covariates), which was consistent with the discovery sample.

## Discussion

In this study, we examined the effects of urban and rural childhood on episodic memory and related brain function in a large and genetically homogeneous sample of young healthy Han Chinese adults. Despite having similar current educational and occupational status, these individuals differed in their urban or rural childhood during China’s rapid and large-scale urbanization. Rural childhood appeared to result in stronger brain activation during encoding, especially in the striatum; and stronger brain activation during retrieval, especially in the hippocampus. These findings were replicated in an independent sample. The different urban–rural striatal emotional encoding functions were associated with the *NTRK2* common variant, which exhibited a higher distribution of the T allele in the urban group. These findings suggest that effects on striatal function may be related to childhood urbanicity, emotional memory processes, and genetic variability within *NTRK2*.

Both groups exhibited reduced accuracy to aversive stimuli during the encoding session, indicating a tendency to avoid aversive stimuli. However, the urban group demonstrated a smaller decrease, which suggests that the urban participants were less likely to neglect aversive stimuli compared to their rural counterparts. The absence of a significant interaction effect between urbanicity and valence in the replication sample may be attributed to the limited sample size, necessitating a more cautious interpretation of behavioral measures. During the retrieval session, episodic memory for aversive stimuli was generally better than for neutral stimuli, consistent with the memorability and ease of recall of aversive stimuli [38]. However, participants with rural childhoods showed less of this effect under aversive stimuli in both cohorts, which may reflect a tendency toward enhanced memory for negative stimuli among urban participants.

During the encoding phase, the rural group showed greater activation in the dorsal striatum than the urban group in both the discovery and replication samples, particularly in response to aversive stimuli. The striatum, especially the caudate nucleus [42], is involved in the formation of declarative memory, the process by which episodic elements are bound to a complete memory trace. One possible explanation for this activation is that networks involved in incremental learning, including the striatum, contribute to the binding process in the formation of integrated episodes [43]. Considering the role of the striatum in processing reward stimuli, the removal of an image may serve as a reward. The lower engagement of the striatum in the urban group may reflect weaker encoding of this positive stimuli. Given that striatal activation has also been associated with aversive learning, a potential reason may be that participants are contemplating how to avoid the potentially negative outcome [44]. In our sample, rural volunteers, when confronted with aversive stimuli, exhibited higher levels of brain activation and lower recall accuracy, aligning with this hypothesis. Furthermore, the higher accuracy demonstrated by the urban group when facing aversive stimuli may suggest a more automatic processing of negative information among adults with an urban upbringing.

During the retrieval phase, the rural group showed greater activation in the hippocampus and parahippocampal gyrus compared to the urban group, particularly in response to aversive stimuli. The hippocampus is involved in emotional memory recall and regulation [45, 46]. While rural participants exhibited increased hippocampal engagement, suggesting a greater investment in memory recall, they still displayed lower accuracy than their urban counterparts when facing aversive stimuli. However, their accuracy levels were similar when encountering neutral stimuli. These results may reflect a protective mechanism of emotional regulation that successfully inhibits negative memory recall.

Previous studies have hypothesized that memory encoding competes for striatal processing in the hippocampus [47, 48]. If this hypothesis holds true, the stronger striatal activation observed in rural participants during encoding could indicate selective neglect of aversive episodic memory through inhibition of hippocampal function. During the retrieval session, despite rural participants displaying increased hippocampal activity, their accuracy remained lower than that of the urban participants. This could be attributed to their selective suppression of the encoding of negative stimuli. Therefore, we suggest that rural participants possess an adaptive striatal function that is less engaged by aversive stimuli, and therefore less affected by or more resilient to stress.

On the other hand, another possibility is that urban participants may more automatically focus on and remember aversive stimuli than their rural counterparts. The pathological process of enhancing negative memories or neglecting positive memories has been observed in conditions including depression, anxiety, and PTSD [8, 10, 49]. A meta-analysis indicated that, when exposed to negative stimuli, patients with depression exhibited lower striatal response levels compared to healthy volunteers, possibly due to decreased striatal dopamine levels when confronted with negative information [50]. Compared to rural volunteers, the brain activation patterns of urban volunteers were analogous to that observed in the depression models. In addition, the reduced prefrontal engagement of urban participants under aversive stimuli may indicate hypersensitivity to negative events, suggesting a potential “sensitizing effect” in urban participants [51].

Our exploratory Genome-Wide Association Studies (GWAS) analysis suggesting genetic variability within *NTRK2* gene affected the striatal processing of aversive stimuli in relation to childhood urbanicity was based on a modest sample of over 400 participants (power = 98.62% calculated by Quanto [52]). *NTRK2* (also known as Tropomyosin receptor kinase B, *TrkB*) is activated by several neurotrophins and serves as a high-affinity receptor for brain-derived neurotrophic factor (BDNF). Although these findings are consistent with previous suggestions that correlations between SNP data and task-related brain imaging data can offer clues about genetic mechanisms [53], some caution is warranted. Firstly, we note that GWAS of complex diseases including in psychiatry, and in psychological constructs (e.g., intelligence quotient, psychological traits) [54, 55] has generally implicated genetic variants with small effect sizes. Our limited sample size thus limits the power to detect additional small genetic and/or environmental effects that could be present, despite the large environmental differences in this unique, genetically homogenous sample, and the relatively large neuroimaging samples herein. For similar reasons, it is also possible that effect sizes here are overestimated. Replication in independent samples is thus needed, which we provide. Here, we again observe the differential striatal activity between two genotype groups, suggesting that the involvement of BDNF signaling in these effects could be robust. Nevertheless, we suggest that future work should be needed in larger populations, and indeed in populations with improved characterization of features in the urban environment (e.g., local-level pollution, density, noise). BDNF expression and downstream signaling through the TrkB receptor are essential for memory formation in aversive domains [56]. BDNF-TrkB signaling within the ventral tegmental area (VTA) - nucleus accumbens (NAc) circuit has also been reported as a pathological mechanism during periods of chronic stress, resulting in depression [57]. Accumulating evidence suggest a relationship between *NTRK2* and a broad range of psychiatric disorders, especially those associated with stress, including depression, schizophrenia, and anxiety disorders. Genotype-dependent differences in *NTRK2* have been observed in white matter properties among patients with depression [58] and have been linked to emotional arousal in healthy individuals [59]. Furthermore, *NTRK2* plays a role in modulating fear learning and synaptic plasticity in the amygdala [60]. Our discovery of *NTRK2* common variations associated with differential urban-rural striatal encoding activities in aversive conditions through genome-wide association analysis supports the potential role of *NTRK2* in emotion dysregulation. The high expression levels of *NTRK2* in the putamen and frontal cortex throughout the lifespan, along with genotype-related expression patterns, suggest its involvement in general neurodevelopmental processes underlying stress and emotion, which may have implications for the mental health of individuals undergoing urban migration.

This study had several limitations. Firstly, the rural group of our subjects was slightly older, and this could potentially have influenced the results, although we did control for age as a factor. Secondly, because our analysis was performed on a population of relatively highly educated individuals in large cities, it may not fully represent people who still reside in rural areas. Further research should aim to include participants with varying socioeconomic statuses, encompassing both patients and healthy individuals, while incorporating variables that could directly reflect early life rural–urban features and considering additional potential confounding factors. Thirdly, our GWAS findings that suggest the involvement of BDNF signaling in the striatal processing of aversive stimuli were exploratory. Although the differential striatal activity had been observed between two genotype groups in the replication sample, more replications were needed in a larger sample. Fourthly, the use age 12 in defining childhood urbanicity is not meant to suggest any empirical evidence that the age of 12 constitutes some critical juncture for the impact of urbanicity on neurodevelopmental processes. Rather, this category variable was selected on the basis that at least in our population context (and many others), age 12 is a convenient demarcation between elementary and middle or secondary school. Moreover, we note that if instead of using such a categorical urbanicity variable, we use a continuous urbanicity variable such as the urbanicity score [17] (Additional file 1: Figure S5), similar results are obtained.

## Conclusions

In conclusion, childhood in rural or urban environments appears to be associated with behavioral and brain physiological differences, particularly in the neural processing of aversive episodic memory within the striatal brain regions. Rural individuals may possess an adaptive striatal function that enhances less aversive memory and inhibits aversive memory. In contrast, urban individuals might have sensitized brain function to negative stress and memory processes. *NTRK2* may play a significant role in the impact of childhood urbanicity on striatal encoding of aversive memory.

### Supplementary information

**Additional file 1: **Supplementary Methods. Figure S1. Principal component analysis of the discovery sample. Figure S2. Activations at different episodic memory conditions. Figure S3. Brain activation of episodic memory task across four groups with different urbanicity. Figure S4. Participants exhibited progressive weaker brain activations with gradual exposure to levels of urbanicity across the four groups. Figure S5. Early-life urbanicity effect on episodic memory task using the urbanicity score. Figure S6. Participants exhibited progressive weaker brain activations with gradual exposure to urbanicity score. Figure S7. Gene expression profiles of *NTRK2* gene and rs3177121. Figure S8. The overlapped brain regions showing the effects of urbanicity in the discovery and replication sample. Table S1. Brain activation during encoding. Table S2. Brain activation during retrieval. Table S3. Rural subjects have more brain activation during encoding session. Table S4. Rural subjects have more brain activation during retrieval session. Table S5. Brain-Behavior correlation using d-prime under the neutral stimulation as the dependent variable. Table S6. Brain-Behavior correlation using recognition accuracy under the neutral stimulation as the dependent variable included socioeconomic status as covariate. Table S7. Brain-Behavior correlations using d-prime under the neutral stimulation as the dependent variable included socioeconomic status as covariate.

## Data Availability

The data that support the findings of this study are available from the corresponding author upon reasonable request.

## Abbreviations

ARL8B: ADP ribosylation factor like GTPase 8 B
BDNF: Brain-derived neurotrophic factor
DLPFC: Dorsal-lateral prefrontal cortex
EFN5A: Ephrin A5
fMRI: Functional magnetic resonance imaging
FWE: Family-wise error rate
GLM: General linear model
GMV: Gray matter volume
GRID1: Glutamate ionotropic receptor delta type subunit 1
MGAT5B: Alpha-1,6-Mannosylglycoprotein 6-Beta-N-Acetylglucosaminyltransferase B
MPFC: Medial prefrontal cortex
MTL: Medial temporal lobe
NAc: Nucleus accumbens
NTRK2: Neurotrophic tyrosine kinase receptor type 2
pACC: Pregenual anterior cingulate cortex
PCA: Principal component analysis
PCC: Posterior cingulate cortex
PFC: Prefrontal cortex
PTSD: Post-traumatic stress disorder
rGE: Gene–environment correlation
ROIs: Regions of interest
SCL25A18: Solute carrier family 25 member 18
SCML4: scm polycomb group protein like 4
SES: Socioeconomic status
SNP: Single-nucleotide polymorphism
TrkB: Tropomyosin receptor kinase B
VTA: Ventral tegmental area
