# Supplementary material for: Childhood urbanicity is associated with emotional episodic memory-related striatal function and common variation in NTRK2
Source: BMC Med. 2024 Apr 2;22:146. doi: 10.1186/s12916-024-03365-4 (PMC10986069; doi:10.1186/s12916-024-03365-4)
Supplement: Supplementary file 1 — Additional file 1: Supplementary Methods. Figure S1. Principal component analysis of the discovery sample. Figure S2. Activations at different episodic memory conditions. Figure S3. Brain activation of episodic memory task across four groups with different urbanicity. Figure S4. Participants exhibited progressive weaker brain activations with gradual exposure to levels of urbanicity across the four groups. Figure S5. Early-life urbanicity effect on episodic memory task using the urbanicity score. Figure S6. Participants exhibited progressive weaker brain activations with gradual exposure to urbanicity score. Figure S7. Gene expression profiles of NTRK2 gene and rs3177121. Figure S8. The overlapped brain regions showing the effects of urbanicity in the discovery and replication sample. Table S1. Brain activation during encoding. Table S2. Brain activation during retrieval. Table S3. Rural subjects have more brain activation during encoding session. Table S4. Rural subjects have more brain activation during retrieval session. Table S5. Brain-Behavior correlation using d-prime under the neutral stimulation as the dependent variable. Table S6. Brain-Behavior correlation using recognition accuracy under the neutral stimulation as the dependent variable included socioeconomic status as covariate. Table S7. Brain-Behavior correlations using d-prime under the neutral stimulation as the dependent variable included socioeconomic status as covariate. [file 12916_2024_3365_MOESM1_ESM.docx]

# Supplementary Methods

## Participants

A total of 522 healthy subjects were recruited from the local community in Beijing and written informed consent was obtained from each subject. We recruited subjects by advertising the study using social media and posters in the community. All participants were assessed by psychiatrists using the Structured Clinical Interview for DSM-IV-TR Axis I Disorders, Research Version, Non-patient Edition (SCID-I/NP) to exclude the history or presence of a psychiatric disorder. Further, subjects met the following inclusion criteria: 18 to 45 years old; right-handed; Chinese of Han ancestry; no history of psychiatric or neurological diseases and substance abuse or dependence; no history of more than 5 minutes’ loss of consciousness; and no abnormalities on subsequent MR images, confirmed by a radiologist.

To determine urbanicity, all subjects provided residence details from birth to the present time. We also ensured that their relocation patterns were exclusively from rural to urban areas, without any return to rural settings. In our study, urbanicity was quantified using inhabitants’ number with Chinese administrative divisions as supplement [31]. The structural hierarchy of the administrative divisions of China includes provincial, prefectural, county and township level. The seat of the provincial, prefectural, and county level government is considered as cities in national census. Township level is the basic level of political divisions in China, and towns usually have less than 10,000 residence inhabitants with several jurisdictional villages surrounded. We define rural environment as administrative towns and villages usually less than 10,000 residence inhabitants. We specifically confirmed with participants that they lived in rural environments with sufficient green space and were predominantly engaged in agricultural cultivation. Urban areas had to have been cities with populations typically more than 100,000 to well over several million. We specifically confirmed with participants that they are in a typical urban environment. Currently, all subjects are living in Beijing (basically located within Beijing's main urban area (the fifth ring road), surrounded by modern urban buildings), which is a super city with more than 20,000,000 inhabitants, and their educational and occupational achievements are similar.

We collected MRI, genetic, and questionnaire data from all subjects. Each dataset was then carefully screened and excluded for (1) incomplete residency information; (2) data quality through visual inspection; (3) head motion (data from participants with head motion greater than 2 mm and/or head rotation greater than 2° were excluded); (4) did not complete the task or knowing the retrieval task before the task; (5) Accuracy in any task less than 50%; (6) did not finish the national nine-year education program. 410 subjects were included in the final analysis.

**Episodic memory paradigm**

Both the encoding and retrieval sessions consisted of 17 blocks (four aversive, four neutral, and nine rest conditions), and each block was preceded by a brief 2-sec instruction screen with a total scan time of 5 min 40 sec. The participants completed the entire encoding session before beginning the retrieval session after a brief delay (about 2 min). In addition, the presentation order of “indoor” and “outdoor” scenes during encoding, “old” and “new” scenes during retrieval, and the aversive and neutral blocks of both sessions were counterbalanced across participants. The subjects answered these questions though pressing the button with their right hand.

**fMRI Acquisition and Statistic Analysis**

Bold fMRI was acquired on a 3.0 T General Electric Discovery MR750 scanner with a gradient-echo, echo-planar imaging sequence in the Center for MRI Research, Peking University. Each functional image consisted of 33 (4.2 mm thick, 0 mm gap) axial slices covering the entire cerebrum and cerebellum (TR/TE = 2000/30 ms, flip angle = 90°, field of view = 24 cm, 64 × 64 matrix). Scanning parameters were selected to optimize the quality of the BOLD signal, and 174 images for the encoding as well as the retrieval sessions were acquired with 4 images as dummy scans. Functional image analysis was completed using Matlab and SPM12 (www.fil.ion.ucl.ac.uk/spm). For each session (encoding and retrieval), subsequent images were realigned to the first image in the series to correct for head motion. These images were then spatially normalized to the MNI template using a fourth-degree B-spline interpolation. Then the images were smoothed using an isotropic 8-mm full-width half-maximum kernel.

For both the encoding and retrieval sessions, we performed a first-level analysis using the General Linear Model (GLM) with six head motions as covariates of no interest. Individual t-contrast maps were generated for both sessions with the following contrasts of interest: aversive > baseline; neutral > baseline; and aversive > neutral (**Figure S2, Table S1 and S2**). Regarding the urbanicity differences in the fMRI task, for those contrasts that did not survive whole-brain family-wise error (FWE) correction, we applied a cluster extent threshold of 100 with a primary threshold of *P* < 0.001. The decision to use 100 voxels was informed by insights drawn from previous published studies [32, 33].

**Brain-Behavior Correlations**

Simple regressions were performed between encoding activation maps and recognition accuracy, with age, sex, and socioeconomic status (SES) as covariates to assess which brain regions were associated with recognition accuracy during encoding. We found that the activation of the right DLPFC (peak at [44 22 42], t = 4.50, cluster size = 356, *P*_cluster-wise_ _FWE_ = 0.035) and the right striatum (peat at [24 12 -6], t = 4.06, cluster size = 517, *P*_cluster-wise_ _FWE_ = 0.009) was associated with neutral retrieval accuracy (Table S6). Specifically, under neutral stimuli, the retrieval accuracy was positively correlated with encoding brain activations of the DLPFC (*P* < 0.001; r = 0.219) and the striatum (*P* < 0.001, r = 0.134) with age, sex, and SES as covariates. A similar result was observed when d-prime was used as the dependent variable (Table S5). We did not find any significant correlations under aversive stimuli.

## DNA Collection and Genotyping

Genomic DNA from the Beijing samples were extracted from peripheral blood using the QIAamp DNA Mini Kit (QIAGEN). Genotyping of samples was conducted using Illumina Human Omni ZhongHua BeadChips, designed for the Chinese population. Normalized bead intensity data obtained for each sample were loaded into Illumina BeadStudio software, which converted fluorescence intensities into SNP genotypes. Samples were excluded (N=15) according to the following quality-control criteria: (1) genotype call rate of <95%, (2) gender discordance, (3) first- or second-degree relatedness, or (4) the genetic outliers. SNPs were excluded using the following criteria: (1) minor allele frequency (MAF) <0.01, (2) genotype call rate of <95%, (3) P values for Hardy-Weinberg equilibrium < 1e-5. Principal Component Analysis (PCA) was performed to identify genetic outliers and determine whether population stratification existed between our urban and rural samples, using EIGENSTRAT (<http://genetics.med.harvard.edu/reich/Reich_Lab/Software.html)>. We compared the first 20 PCAs among the two urbanicity groups using a two-sample t-test with statistical significance set at *P*<0.05 corrected for the number of independent components tested.

# Supplementary Figures

## Figure S1 Principal component analysis of the discovery sample.

We compared the first 20 PCAs and there were no significant differences across the first 20 principal components from whole genome genotyping. This figure showed the first and second principal component.

## Figure S2 Activations at different episodic memory conditions in the discovery sample.


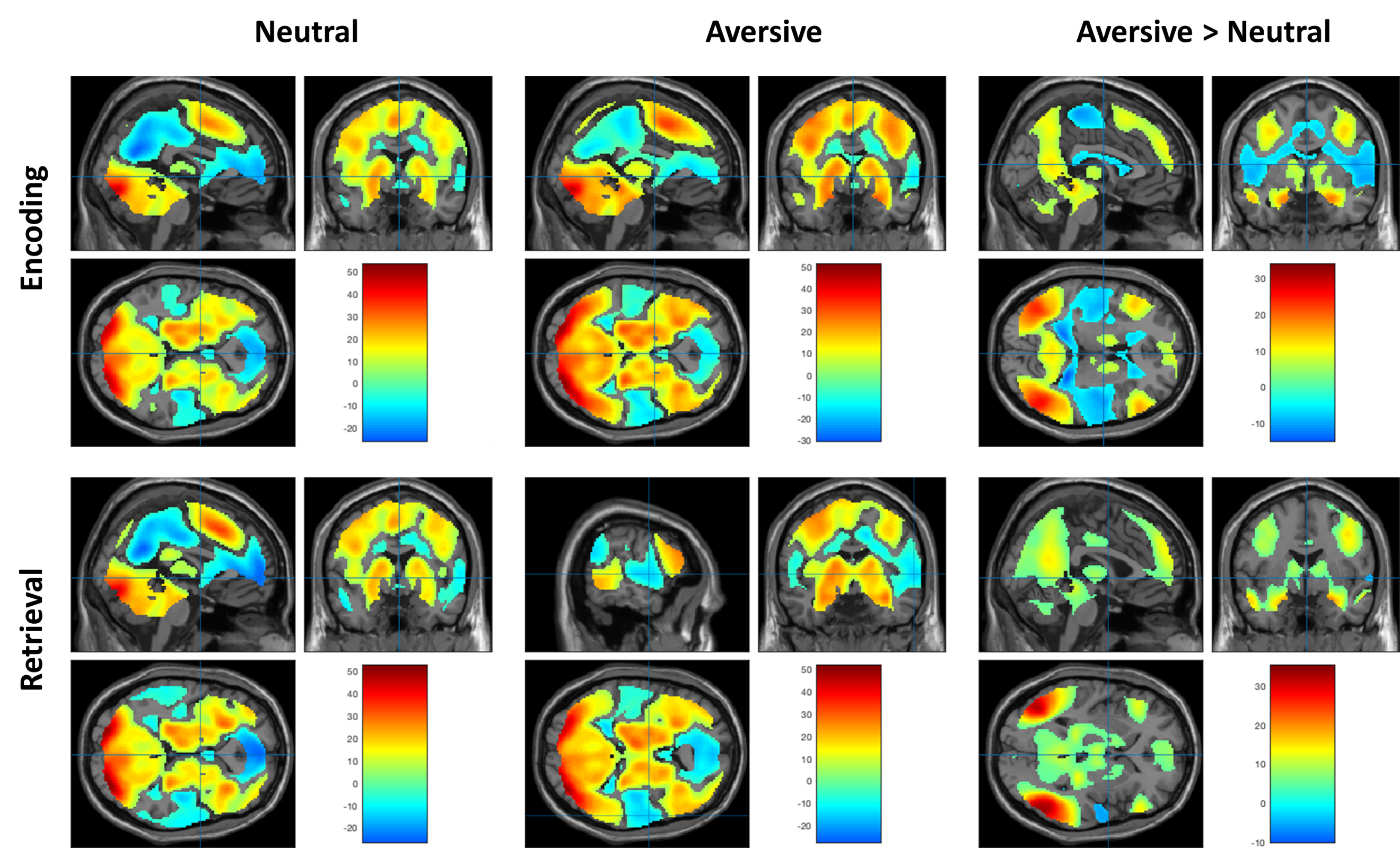


Left and Middle Column: During the encoding/retrieval session under both neutral and aversive stimuli, regions in the dorsolateral prefrontal cortex, occipital visual cortex, parts of temporal and parietal lobe, hippocampus, striatum and amygdala were robustly engaged, while there was decreased engagement of parts of medial prefrontal cortex, posterior cingulate cortex, insula and precuneus (*P* <0.05 FWE whole brain corrected, cluster > 100).

Right Column: During the encoding session, the aversive task exhibited increased bilateral activation in regions such as the occipital gyrus, inferior frontal lobe, hippocampus, thalamus, medial prefrontal cortex, posterior cingulate cortex, etc., as compared to the neutral task. Additionally, there was decreased bilateral activation observed in the insula and postcentral gyrus (*P* <0.05 FWE whole brain corrected, cluster > 100). During the retrieval session, the aversive task exhibited increased bilateral activation in regions such as the occipital gyrus, inferior frontal lobe, hippocampus, thalamus, medial prefrontal cortex, posterior cingulate cortex, etc., as compared to the neutral task. Additionally, there was decreased activation observed in the right fusiform and right superior temporal gyrus (*P* <0.05 FWE whole brain corrected, cluster > 100).

## Figure S3 Brain activation of episodic memory task across four groups with different urbanicity in the discovery sample.


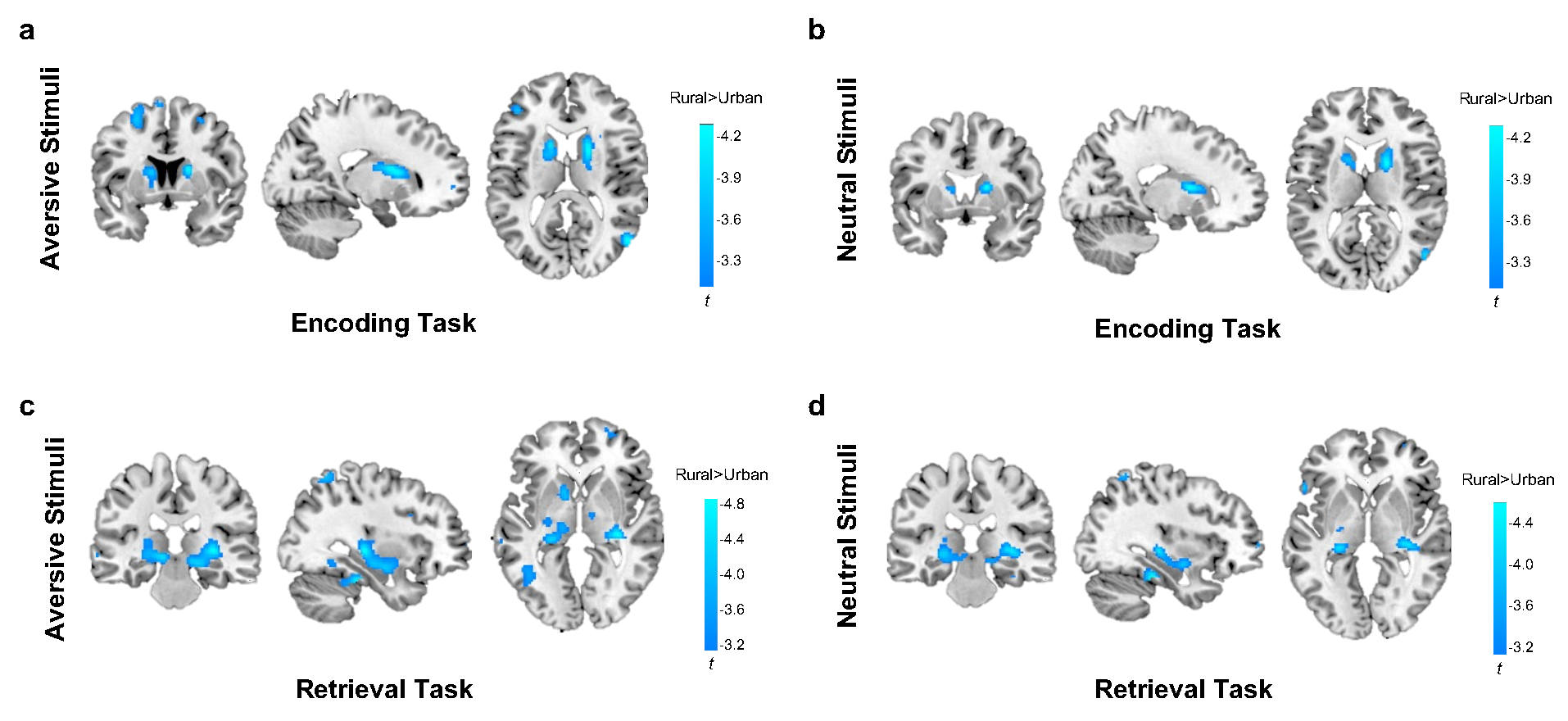


The four groups were defined as follows: Group #1: individuals who were born in and continue to live in cities; #2: individuals who have lived in cities since before age 12; #3: individuals who were born in and continue to live in rural areas until age 12-18; #4: individuals lived in rural areas for ≥18 years since birth). (a) During the encoding session, a positive correlation was observed between increased rural experience and heightened activation in the bilateral caudate and putamen, as well as the bilateral middle frontal gyrus, in response to aversive stimuli (shown at *P* < 0.001, peak atlas could withstand whole-brain cluster-wise FWE-corrected, *P* < 0.05). (b) During the encoding session, a positive correlation was observed between increased rural experience and heightened activation in right caudate; and left putamen and inferior temporal lobe under neutral stimuli (shown at *P* < 0.001). (c) During the retrieval session, a positive correlation was observed between increased rural experience and heightened activation in bilateral middle temporal gyrus; bilateral fusiform and thalamus under aversive stimuli (shown at *P* < 0.001, peak atlas could withstand whole-brain FWE-corrected, *P* < 0.05). (d) During the retrieval session, a positive correlation was observed between increased rural experience and heightened activation in bilateral fusiform; left hippocampus and amygdala under neutral stimuli (shown at *P* < 0.001, peak atlas could withstand whole-brain FWE-corrected, *P* < 0.05).

## Figure S4 Participants exhibited progressive weaker brain activations with gradual exposure to levels of urbanicity across the four groups in the discovery sample.


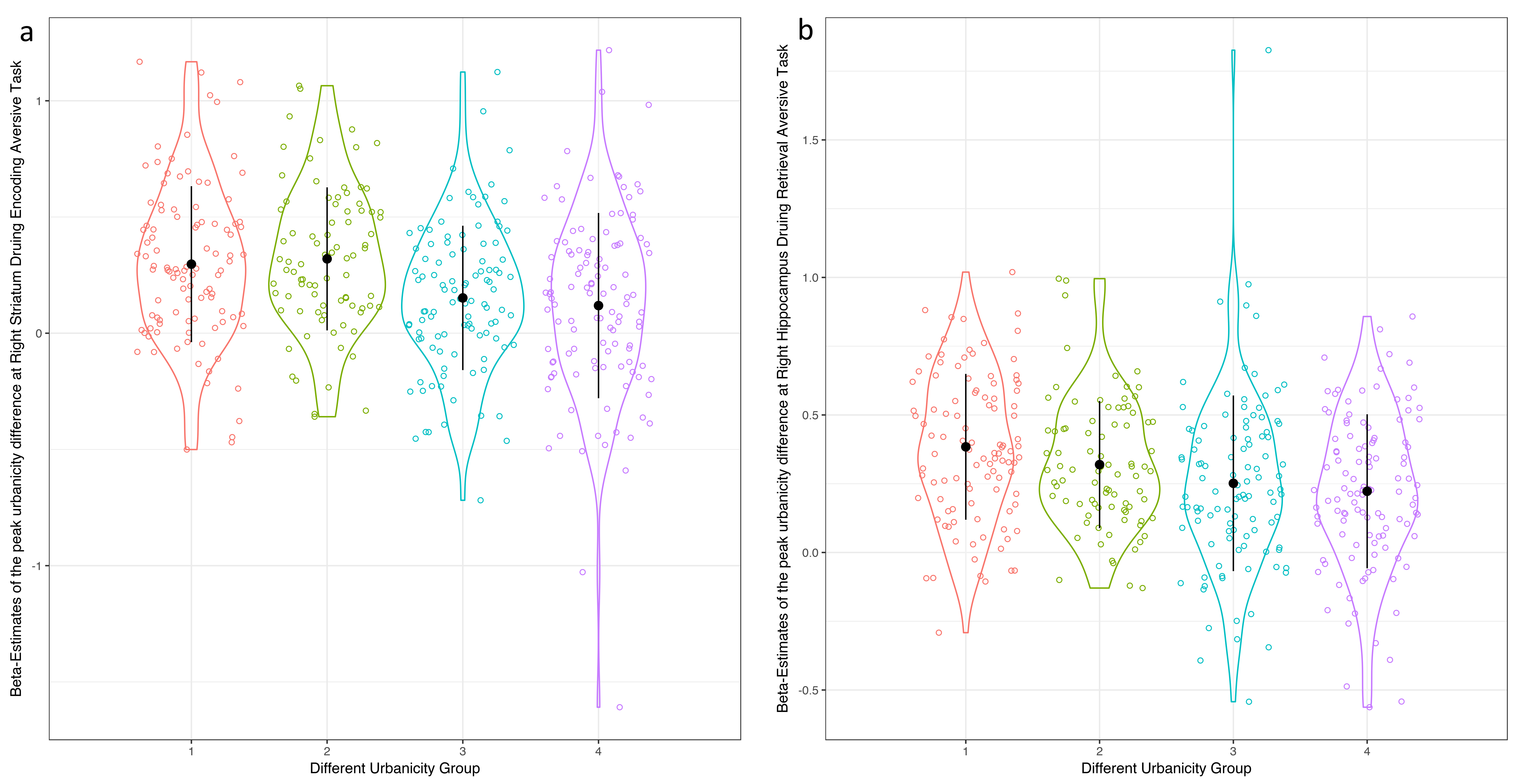


(a) During the encoding session, participants exhibited progressive weaker brain activations with gradual exposure to levels of urbanicity across the four group, exemplified by the right striatum (peak at [18 6 14]). (b) During the retrieval session, participants exhibited progressive weaker brain activations with gradual exposure to levels of urbanicity across the four group, exemplified by the right hippocampus (peak at [34 -10 -14]).

## Figure S5 Early-life urbanicity effect on episodic memory task using the urbanicity score in the discovery sample.


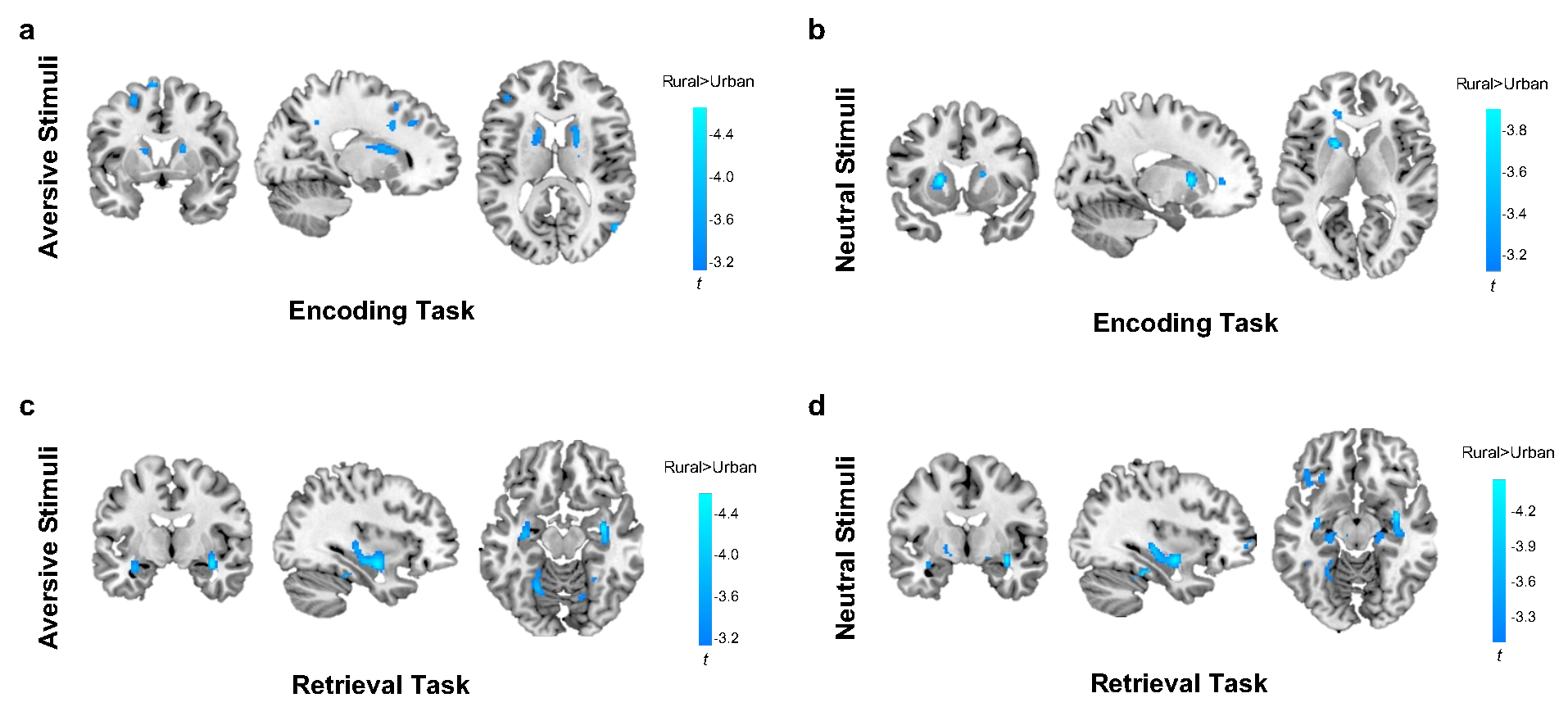


The urbanicity score was defined according to population size as follows: population < 10000 = 1, less than 1,000,000 residents = 2; cities with more than 1,000,000 residents = 3; the category scores were then multiplied by the number of years spent in the location until age 15. (a) During the encoding session, a positive correlation was observed between increased rural experience and heightened activation in the bilateral caudate and putamen, right middle temporal gyrus, as well as the bilateral middle frontal gyrus, in response to aversive stimuli (shown at *P* < 0.001, peak atlas could withstand whole-brain FWE-corrected, *P* < 0.05). (b) During the encoding session, a positive correlation was observed between increased rural experience and heightened activation in left putamen under neutral stimuli (shown at *P* < 0.001). (c) During the retrieval session, a positive correlation was observed between increased rural experience and heightened activation in bilateral hippocampus; right middle temporal gyrus under aversive stimuli (shown at *P* < 0.001, peak atlas could withstand whole-brain cluster-wise FWE-corrected, *P* < 0.05). (d) During the retrieval session, a positive correlation was observed between increased rural experience and heightened activation in bilateral hippocampus; right fusiform; left thalamus and amygdala under neutral stimuli (shown at *P* < 0.001, peak atlas could withstand whole-brain cluster-wise FWE-corrected, *P* < 0.05).

## Figure S6 Participants exhibited progressive weaker brain activations with gradual exposure to urbanicity score in the discovery sample.


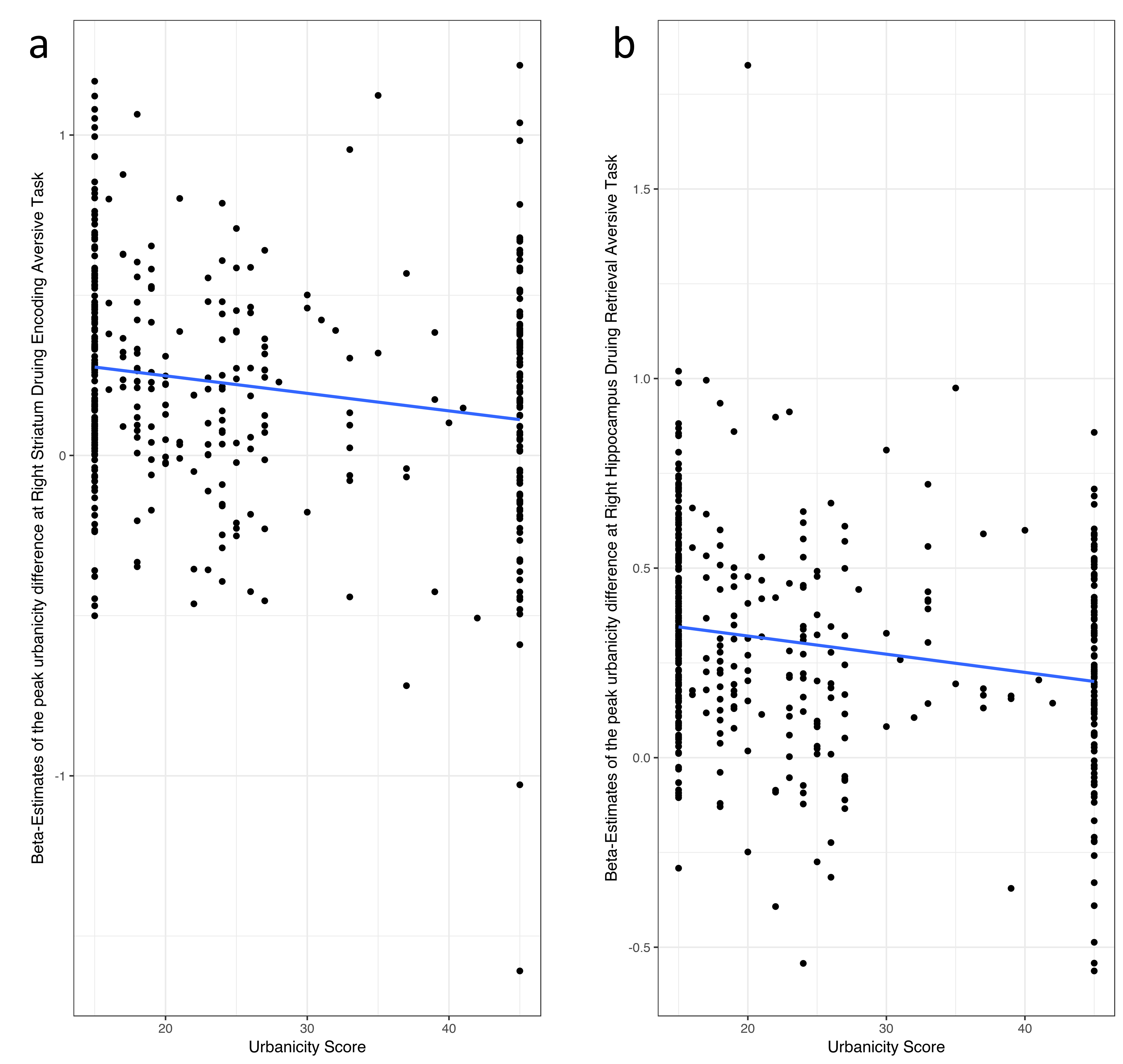


(a) During the encoding session, participants displayed a negative correlation between brain activations and urbanicity score, exemplified by the right caudate (peak at [18 6 14], *P* < 0.001, r = -0.19). (b) During the retrieval session, participants displayed a negative correlation between brain activations and urbanicity score, exemplified by the right hippocampus (peak at [34 -10 -14], *P* < 0.001, r = -0.21).

## Figure S7 Gene expression profiles of *NTRK2* gene and rs3177121.


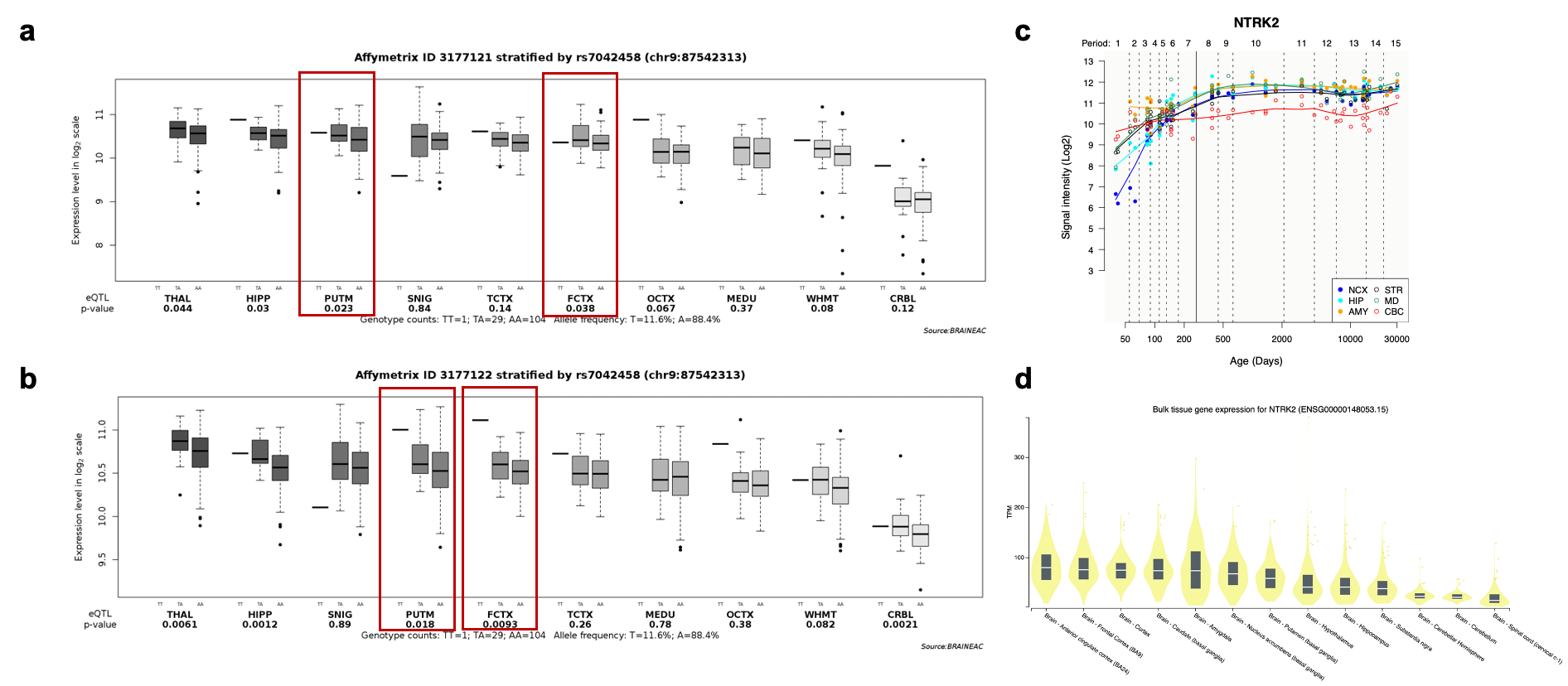


(a, b) Public expression data from Brainace Database indicated that *NTRK2* common variant rs7042458 affected gene expression in putamen and frontal cortex, in which AT/TT group exhibited higher expression. (c) Spatiotemporal gene expression for NTRK2. (d) Bulk tissue gene expression for NTRK2.

## Figure S8 The overlapped brain regions showing the effects of urbanicity in the encoding memory task in response to aversive stimuli in the discovery and replication sample.


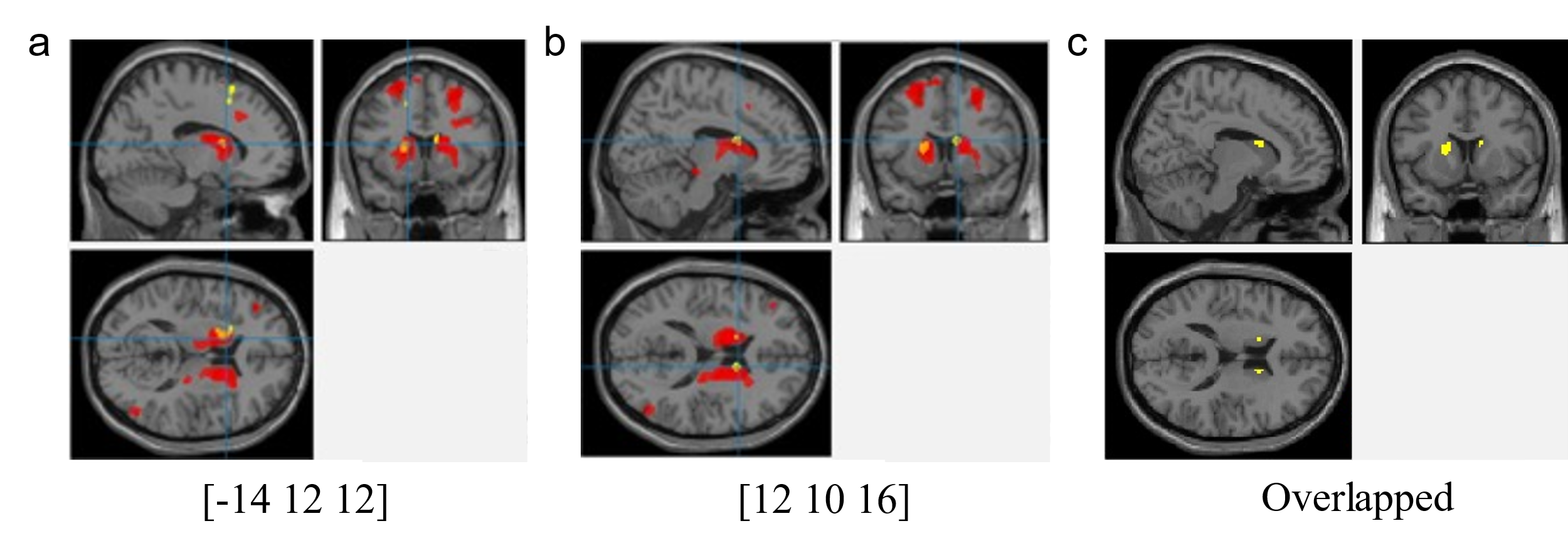


(a), the overlapped left caudate and putamen with cross in the [-14 12 12]; (b), the overlapped right caudate with cross in the [12 10 16]; (c), the overview of the overlapped brain regions including bilateral caudate and left putamen. Red, brain regions showing the effects of urbanicity in the encoding memory task in response to aversive stimuli in the discovery sample; yellow, brain regions showing the effects of urbanicity in the encoding memory task in response to aversive stimuli in the replication sample.

# Supplementary Tables

## Table S1 Brain activation during encoding in the discovery sample.

| **Peak Atlas** | **Cluster** | **x** | **y** | **z** | **T** |
| --- | --- | --- | --- | --- | --- |
| **Encoding: Aversive - Rest** | | | | | |
| L Medial Frontal Gyrus | 81021 | -4 | 8 | 52 | 34.19 |
| L Insula |  | -32 | 26 | -4 | 27.70 |
| R Insula |  | 34 | 26 | -4 | 24.96 |
| R Middle Frontal Gyrus |  | 52 | 32 | 18 | 33.55 |
| L Inferior Frontal Gyrus |  | -54 | 26 | 22 | 30.01 |
| L Inferior Frontal Gyrus |  | -44 | 8 | 28 | 33.96 |
| R Middle Frontal Gyrus |  | 48 | 14 | 30 | 36.91 |
| L Middle Frontal Gyrus |  | -52 | 26 | 42 | 22.29 |
| L Insula |  | -32 | 26 | -4 | 27.70 |
| L Lentiform Nucleus |  | -16 | -2 | 10 | 24.57 |
| R Cerebrum |  | 16 | 4 | 8 | 22.15 |
| L Hippocampus |  | -24 | -30 | -8 | 37.71 |
| R Hippocampus |  | 24 | -28 | -8 | 39.42 |
| L Medial Frontal Gyrus |  | -8 | 26 | -12 | 33.75 |
| R Midbrain |  | 8 | -26 | -12 | 31.70 |
| L Culmen |  | -30 | -52 | -24 | 42.60 |
| R Declive |  | 38 | -58 | -26 | 38.87 |
| L Middle Occipital Gyrus |  | -26 | -90 | -2 | 47.89 |
| R Sub-Gyral |  | 26 | -88 | -4 | 49.24 |
| L Precuneus |  | -24 | -66 | 40 | 27.07 |
| R Precuneus |  | 28 | -68 | 40 | 25.98 |
|  |  |  |  |  |  |
| **Encoding: Neutral - Rest** | | | | | |
| L Medial Frontal Gyrus | 77438 | -4 | 6 | 52 | 31.26 |
| L Insula |  | -32 | 22 | -2 | 21.15 |
| R Insula |  | 34 | 24 | -2 | 20.50 |
| R Middle Frontal Gyrus |  | 52 | 36 | 20 | 26.15 |
| L Inferior Frontal Gyrus |  | -52 | 10 | 36 | 29.61 |
| L Inferior Frontal Gyrus |  | -46 | 46 | 2 | 20.82 |
| R Middle Frontal Gyrus |  | 48 | 14 | 30 | 31.10 |
| L Insula |  | -32 | 22 | -2 | 21.15 |
| R Insula |  | 34 | 24 | -2 | 20.50 |
| L Lentiform Nucleus |  | -24 | -4 | 2 | 26.47 |
| R Lentiform Nucleus |  | 22 | -2 | 4 | 19.65 |
| L Thalamus |  | -14 | -20 | 2 | 26.52 |
| R Extra-Nuclear |  | 24 | -28 | -8 | 37.20 |
| L Extra-Nuclear |  | -24 | -30 | -8 | 35.16 |
| R Midbrain |  | 8 | -24 | -14 | 26.49 |
| L Midbrain |  | -10 | -22 | -12 | 27.53 |
| L Culmen |  | -32 | -48 | -26 | 41.01 |
| R Culmen |  | 32 | -44 | -26 | 45.52 |
| L Middle Occipital Gyrus |  | -26 | -90 | -2 | 46.03 |
| R Lingual Gyrus |  | 22 | -92 | 0 | 46.90 |
| R Lingual Gyrus |  | 8 | -82 | -12 | 53.18 |
| L Precuneus |  | -24 | -64 | 36 | 23.25 |
| R Precuneus |  | 30 | -66 | 36 | 22.71 |
| L Postcentral Gyrus |  | -58 | -22 | 48 | 26.26 |
| R Postcentral Gyrus |  | 60 | -14 | 24 | 10.54 |
|  |  |  |  |  |  |
| **Encoding: Aversive - Neutral** | | | | | |
| L Medial Frontal Gyrus | 45825 | -2 | 52 | 30 | 9.95 |
| R Superior Frontal Gyrus |  | 2 | 18 | 56 | 9.71 |
| R Inferior Frontal Gyrus |  | 54 | 30 | 18 | 16.78 |
| R Inferior Frontal Gyrus |  | 44 | 10 | 30 | 18.92 |
| L Extra-Nuclear |  | -12 | 4 | 8 | 9.26 |
| R Extra-Nuclear |  | 12 | 6 | 8 | 8.82 |
| L Amygdala |  | -22 | -4 | -22 | 17.56 |
| R Amygdala |  | 24 | -4 | -20 | 18.04 |
| L Hippocampus |  | -22 | -30 | -8 | 12.26 |
| R Hippocampus |  | 20 | -30 | -6 | 14.24 |
| L Culmen |  | -42 | -50 | -28 | 26.52 |
| R Fusiform Gyrus |  | 42 | -52 | -24 | 24.96 |
| L Middle Occipital Gyrus |  | -46 | -76 | -4 | 33.69 |
| R Inferior Temporal Gyrus |  | 48 | -70 | -6 | 33.80 |
| L Hippocampus |  | -24 | -24 | -12 | 12.10 |
| L Midbrain |  | -6 | -28 | -12 | 13.75 |
| R Midbrain |  | 8 | -28 | -12 | 14.45 |
| R Calcarine |  | 18 | -52 | 10 | 9.31 |
| L Posterior Cingulate |  | -4 | -54 | 12 | 11.82 |
| L Superior Parietal Lobule |  | -26 | -56 | 44 | 13.82 |
| R Precuneus |  | 6 | -56 | 44 | 11.03 |
| R Sub-Gyral |  | 26 | -56 | 42 | 16.88 |
| L Inferior Frontal Gyrus | 6215 | -42 | 6 | 30 | 15.47 |
| L Inferior Frontal Gyrus |  | -46 | 22 | 20 | 14.63 |
| L Middle Temporal Gyrus |  | -54 | -2 | -20 | 12.51 |
|  |  |  |  |  |  |
| **Encoding: Neutral - Aversive** | | | | | |
| L Lateral Ventricle | 23154 | -14 | 20 | 16 | 7.89 |
| R Lateral Ventricle |  | 18 | 26 | 14 | 8.60 |
| L Lateral Ventricle |  | -4 | 2 | 18 | 7.25 |
| L Medial Frontal Gyrus |  | -4 | -28 | 60 | 11.18 |
| L Precentral Gyrus |  | -22 | -24 | 64 | 8.26 |
| R Medial Frontal Gyrus |  | 6 | -24 | 62 | 10.90 |
| L Superior Temporal Gyrus |  | -54 | -6 | 0 | 9.80 |
| L Insula |  | -44 | -12 | 16 | 9.64 |
| L Extra-Nuclear |  | -22 | -8 | 26 | 8.32 |
| R Extra-Nuclear |  | 24 | -28 | 26 | 8.91 |
| R Insula |  | 42 | -12 | 16 | 10.94 |
| R Superior Temporal Gyrus |  | 60 | -10 | 0 | 10.04 |
| L Lateral Ventricle |  | -20 | -44 | 10 | 13.27 |
| R Lateral Ventricle |  | 22 | -40 | 10 | 14.71 |
| R Inferior Parietal Lobule |  | 54 | -52 | 44 | 9.97 |
| L Inferior Parietal Lobule | 492 | -50 | -54 | 46 | 7.98 |
| R Cuneus | 117 | 14 | -94 | 0 | 6.92 |
| L Lingual Gyrus |  | -6 | -90 | -10 | 4.63 |
|  |  |  |  |  |  |

N = 410, age as covariates, whole-brain FWE correction, cluster size > 100

## Table S2 Brain activation during retrieval in the discovery sample.

| **Peak Atlas** | **Cluster** | **x** | **y** | **z** | **T** |
| --- | --- | --- | --- | --- | --- |
| **Retrieval: Aversive - Baseline** | | | | | |
| L Medial Frontal Gyrus | 85550 | -4 | 10 | 50 | 33.60 |
| L Middle Frontal Gyrus |  | -34 | 56 | 14 | 19.86 |
| R Middle Frontal Gyrus |  | 36 | 56 | 14 | 18.68 |
| R Middle Frontal Gyrus |  | 52 | 32 | 20 | 31.32 |
| L Inferior Frontal Gyrus |  | -52 | 26 | 22 | 28.93 |
| L Middle Frontal Gyrus |  | -48 | 10 | 34 | 33.79 |
| R Middle Frontal Gyrus |  | 48 | 14 | 30 | 37.85 |
| L Sub-Gyral |  | -14 | -2 | 58 | 29.32 |
| R Middle Frontal Gyrus |  | 44 | 6 | 54 | 26.48 |
| L Lentiform Nucleus |  | -16 | -2 | 10 | 27.45 |
| R Extra-Nuclear |  | 16 | 4 | 10 | 25.90 |
| L Thalamus |  | -12 | -18 | 4 | 29.66 |
| R Thalamus |  | 10 | -4 | 6 | 24.06 |
| R Hippocampus |  | 24 | -30 | -8 | 45.68 |
| L Hippocampus |  | -26 | -28 | -10 | 43.62 |
| R Midbrain |  | 8 | -26 | -12 | 35.76 |
| L Midbrain |  | -8 | -26 | -12 | 36.76 |
| L Culmen |  | -32 | -50 | -26 | 43.99 |
| R Culmen |  | 32 | -44 | -28 | 46.07 |
| L Inferior Occipital Gyrus |  | -36 | -82 | -10 | 46.50 |
| R Middle Occipital Gyrus |  | 28 | -88 | -2 | 51.01 |
| R Lingual Gyrus |  | 8 | -82 | -12 | 51.43 |
| L Superior Parietal Lobule |  | -30 | -60 | 46 | 29.94 |
| R Inferior Parietal Lobule |  | 32 | -60 | 44 | 28.97 |
| L Inferior Parietal Lobule |  | -46 | -34 | 42 | 21.26 |
| R Inferior Parietal Lobule |  | 48 | -36 | 44 | 9.38 |
|  | | | | | |
| **Retrieval: Neutral - Baseline** | | | | | |
| L Medial Frontal Gyrus | 75859 | -2 | 16 | 48 | 33.21 |
| L Inferior Frontal Gyrus |  | -46 | 46 | 2 | 20.37 |
| R Middle Frontal Gyrus |  | 36 | 56 | 12 | 19.89 |
| L Insula |  | -30 | 26 | -4 | 30.87 |
| R Insula |  | 32 | 26 | -4 | 29.92 |
| L Inferior Frontal Gyrus |  | -50 | 10 | 34 | 30.29 |
| R Middle Frontal Gyrus |  | 46 | 12 | 30 | 32.24 |
| R Middle Frontal Gyrus |  | 52 | 32 | 28 | 31.17 |
| L Extra-Nuclear |  | -18 | -2 | 12 | 23.80 |
| R Extra-Nuclear |  | 16 | 4 | 10 | 21.16 |
| L Extra-Nuclear |  | -26 | 2 | -8 | 21.29 |
| R Extra-Nuclear |  | 26 | 6 | -10 | 17.39 |
| L Thalamus |  | -14 | -20 | 4 | 26.29 |
| L Hippocampus |  | -24 | -30 | -8 | 37.66 |
| R Hippocampus |  | 26 | -28 | -8 | 38.40 |
| L Midbrain |  | -8 | -24 | -14 | 27.12 |
| R Midbrain |  | 8 | -26 | -14 | 25.80 |
| L Culmen |  | -32 | -48 | -26 | 42.88 |
| R Culmen |  | 32 | -42 | -28 | 46.25 |
| L Sub-Gyral |  | -24 | -90 | -4 | 51.18 |
| R Sub-Gyral |  | 26 | -88 | -4 | 51.76 |
| R Lingual Gyrus |  | 10 | -84 | -12 | 52.37 |
| L Precuneus |  | -26 | -66 | 40 | 28.19 |
| R Precuneus |  | 30 | -66 | 38 | 27.38 |
| L Inferior Parietal Lobule |  | -44 | -32 | 42 | 20.99 |
| R Inferior Parietal Lobule |  | 48 | -40 | 44 | 10.71 |
|  | | | | | |
| **Retrieval: Aversive - Neutral** | | | | | |
| L Superior Frontal Gyrus | 60307 | -4 | 54 | 28 | 13.92 |
| R Anterior Cingulate |  | 4 | 40 | 4 | 6.07 |
| L Superior Frontal Gyrus |  | -6 | 32 | 58 | 9.41 |
| L Inferior Frontal Gyrus |  | -52 | 30 | 10 | 14.64 |
| L Precentral Gyrus |  | -44 | 0 | 32 | 9.30 |
| R Inferior Frontal Gyrus |  | 52 | 32 | 10 | 16.92 |
| R Inferior Frontal Gyrus |  | 42 | 18 | 24 | 15.13 |
| R Middle Frontal Gyrus |  | 38 | 0 | 44 | 14.74 |
| L Precentral Gyrus |  | -38 | -6 | 44 | 10.72 |
| L Inferior Frontal Gyrus |  | -40 | 28 | -18 | 15.69 |
| R Inferior Frontal Gyrus |  | 42 | 30 | -16 | 10.10 |
| L Caudate |  | -12 | 4 | 10 | 9.10 |
| R Caudate |  | 12 | 0 | 10 | 8.20 |
| L Middle Temporal Gyrus |  | -54 | -4 | -20 | 15.96 |
| R Middle Temporal Gyrus |  | 52 | -6 | -14 | 9.10 |
| L Amygdala |  | -22 | -4 | -20 | 20.27 |
| R Hippocampus |  | 22 | -2 | -22 | 20.67 |
| L Parahippocampa Gyrus |  | -18 | -32 | -8 | 14.13 |
| R Lingual |  | 18 | -30 | -6 | 15.40 |
| L Lingual |  | -16 | -54 | -10 | 7.48 |
| R Fusiform |  | 22 | -58 | -12 | 8.19 |
| L Culmen |  | -42 | -50 | -28 | 26.26 |
| R Fusiform |  | 44 | -52 | -22 | 24.39 |
| L Inferior Temporal Gyrus |  | -48 | -74 | -6 | 33.42 |
| R Inferior Temporal Gyrus |  | 50 | -68 | -6 | 35.14 |
| L Posterior Cingulate |  | -4 | -54 | 20 | 14.04 |
| L Superior Parietal Lobule |  | -26 | -54 | 46 | 10.62 |
| R Superior Parietal Lobule |  | 28 | -54 | 46 | 14.47 |
| L Middle Cingulate |  | -4 | -14 | 36 | 7.49 |
| L Thalamus |  | -6 | -10 | 0 | 10.08 |
| R Thalamus |  | 6 | -10 | 2 | 9.80 |
|  |  |  |  |  |  |
| **Retrieval: Neutral - Aversive** | | | | | |
| R Lateral Ventricle | 228 | 22 | -42 | 10 | 9.94 |
| L Lateral Ventricle | 253 | -20 | -44 | 10 | 9.08 |
| R Sub-Gyral | 160 | 34 | -44 | 10 | 6.43 |
| R Fusiform Gyrus |  | 30 | -42 | -18 | 6.38 |
| R Superior Temporal Gyrus | 255 | 58 | -6 | 0 | 5.84 |
|  |  |  |  |  |  |

N = 410, age and sex were used as covariates, whole-brain FWE correction, cluster size > 100

## Table S3 Rural subjects have more brain activation greater than urban subjects during encoding session in the discovery sample.

| **Peak Atlas** | **Cluster** | **x** | **y** | **z** | **T** |
| --- | --- | --- | --- | --- | --- |
| **Encoding: Aversive – Baseline** | | | | | |
| R Caudate | 3562 | 16 | 12 | 12 | 5.2* |
| R Caudate |  | 18 | 2 | 16 | 5.01* |
| R Middle Frontal Gyrus |  | 24 | 32 | 38 | 4.95* |
| L Caudate | 1695 | -14 | 12 | 12 | 4.98* |
| L Caudate |  | -14 | 2 | 16 | 4.79* |
| L Putamen |  | -22 | 16 | -8 | 4.15 |
| L Superior Frontal Gyrus | 370 | -26 | 10 | 66 | 4.33 |
| L Middle Frontal Gyrus |  | -24 | 6 | 56 | 4.19 |
| L Superior Frontal Gyrus |  | -6 | 10 | 68 | 4.1 |
| R White Matter | 221 | 30 | -20 | -6 | 4.25 |
| R Midbrain |  | 12 | -28 | -10 | 4.01 |
| R Hippocampus |  | 22 | -18 | -16 | 3.45 |
| L White Matter | 283 | -38 | -58 | 2 | 4.21 |
| L Fusiform Gyrus |  | -44 | -58 | -22 | 3.17 |
| L Middle Frontal Gyrus | 166 | -40 | 26 | 28 | 4.01 |
| L Middle Frontal Gyrus |  | -48 | 22 | 32 | 3.66 |
| R Precentral Gyrus | 133 | 54 | -10 | 24 | 3.86 |
| R Middle Temporal Gyrus | 107 | 48 | -70 | 10 | 3.83 |
| L Precentral Gyrus | 168 | -46 | -18 | 56 | 3.79 |
| **Encoding: Neutral – Baseline** | | | | | |
| R Caudate | 154 | 22 | 16 | 10 | 3.92 |

N = 410, age and sex were used as covariates, *P* < 0.001 uncorrected, cluster size > 100

* peak atlas that could withstand whole-brain few correction *P* < 0.05

## Table S4 Rural subjects have more brain activation greater than urban subjects during retrieval session in the discovery sample.

| **Peak Atlas** | **Cluster** | **x** | **y** | **z** | **T** |
| --- | --- | --- | --- | --- | --- |
| **Retrieval: Aversive - Baseline** | | | | | |
| R Middle Temporal Gyrus | 153 | 46 | -72 | 10 | 4.40 |
| R White Matter | 581 | 34 | -8 | -14 | 4.36 |
| R Hippocampus |  | 18 | -24 | -10 | 4.06 |
| R White Matter |  | 30 | -26 | 0 | 4.05 |
| R Fusiform Gyrus | 206 | 36 | -32 | -22 | 4.31 |
| R Fusiform Gyrus |  | 28 | -50 | -14 | 3.98 |
| L Fusiform Gyrus | 185 | -24 | -56 | -12 | 4.30 |
| L Fusiform Gyrus |  | -20 | -46 | -16 | 3.41 |
| L Thalamus | 526 | -24 | -30 | 6 | 4.29 |
| L Midbrain |  | -12 | -26 | -4 | 4.08 |
| L Lentiform Nucleus |  | -26 | -14 | -8 | 4.01 |
| R Middle Frontal Gyrus | 103 | 38 | 14 | 32 | 3.99 |
| L Middle Occipital Gyrus | 453 | -38 | -60 | 4 | 3.95 |
| L Fusiform Gyrus |  | -46 | -56 | -22 | 3.80 |
| L White Matter |  | -40 | -52 | -8 | 3.78 |
| L Cuneus | 125 | -26 | -80 | 8 | 3.71 |
| L Cuneus |  | -26 | -88 | 20 | 3.31 |
| **Retrieval: Neutral - Baseline** | | | | | |
| L Thalamus | 134 | -24 | -28 | -2 | 4.11 |
| L Fusiform Gyrus | 213 | -26 | -54 | -12 | 4.07 |
| L Fusiform Gyrus |  | -22 | -44 | -16 | 3.51 |

N = 410, age and sex were used as covariates, *P* < 0.001 uncorrected, cluster size > 100

## Table S5 Brain-Behavior Correlation using d-prime under the neutral stimulation as the dependent variable with age and sex as covariates in the discovery sample.

| **Peak Brain Region** | **Cluster** | **x** | **y** | **z** | **T** | ***P_peak-FWE_*** | ***P_cluster-wise FWE_*** |
| --- | --- | --- | --- | --- | --- | --- | --- |
| R Middle Frontal Gyrus | 386 | 46 | 24 | 44 | 4.71 | 0.023 | 0.027 |
|  |  | 38 | 14 | 30 | 3.74 | 0.534 |  |
| R Cerebellum Posterior Lobe | 352 | -2 | -76 | -20 | 4.58 | 0.038 | 0.036 |
|  |  | 8 | -70 | -22 | 3.93 | 0.342 |  |
|  |  | 12 | -80 | -28 | 3.71 | 0.563 |  |
| R Putamen | 956 | 24 | 12 | -6 | 4.40 | 0.077 | <0.001 |
|  |  | 18 | -2 | 10 | 4.24 | 0.132 |  |
|  |  | 30 | -16 | -14 | 4.20 | 0.150 |  |

**Table S6 Brain-Behavior Correlations using recognition accuracy under the neutral stimulation as the dependent variable with age, sex, and socioeconomic status as covariates in the discovery sample.**

| **Peak Brain Region** | **Cluster** | **x** | **y** | **z** | **T** | ***P_peak-FWE_*** | ***P_cluster-wise FWE_*** |
| --- | --- | --- | --- | --- | --- | --- | --- |
| R Middle Frontal Gyrus | 356 | 44 | 22 | 42 | 4.50 | 0.053 | 0.035 |
|  |  | 36 | 14 | 28 | 3.84 | 0.423 |  |
| R Putamen | 517 | 24 | 12 | -6 | 4.06 | 0.232 | 0.009 |
|  |  | 20 | -4 | 10 | 3.95 | 0.320 |  |
|  |  | 30 | -14 | -14 | 3.85 | 0.418 |  |

**Table S7 Brain-Behavior correlations using d-prime under the neutral stimulation as the dependent variable with age, sex, and socioeconomic status as covariates in the discovery sample.**

| **Peak Brain Region** | **Cluster** | **x** | **y** | **z** | **T** | ***P_peak-FWE_*** | ***P_cluster-wise FWE_*** |
| --- | --- | --- | --- | --- | --- | --- | --- |
| R Middle Frontal Gyrus | 360 | 46 | 24 | 44 | 4.62 | 0.033 | 0.033 |
|  |  | 36 | 16 | 30 | 3.66 | 0.618 |  |
| R Putamen | 836 | 24 | 12 | -6 | 4.33 | 0.097 |  |
|  |  | 30 | -16 | -14 | 4.21 | 0.146 | 0.001 |
|  |  | 18 | -2 | 10 | 4.15 | 0.175 |  |
